# Downstream-of-gene (DoG) transcripts contribute to an imbalance in the cancer cell transcriptome

**DOI:** 10.1126/sciadv.adh9613

**Published:** 2024-07-03

**Authors:** Kouki Abe, Brian Maunze, Pedro-Avila Lopez, Jessica Xu, Nefertiti Muhammad, Guang-Yu Yang, David Katz, Yaping Liu, Shannon M. Lauberth

**Affiliations:** ^1^ Simpson Querrey Institute for Epigenetics, Feinberg School of Medicine, Northwestern University, Chicago, IL 60611, USA.; ^2^ Department of Biochemistry and Molecular Genetics, Feinberg School of Medicine, Northwestern University, Chicago, IL 60611, USA.; ^3^ Department of Pathology, Northwestern University Feinberg School of Medicine, Chicago, IL 60611, USA.

## Abstract

Downstream-of-gene (DoG) transcripts are an emerging class of noncoding RNAs. However, it remains largely unknown how DoG RNA production is regulated and whether alterations in DoG RNA signatures exist in major cancers. Here, through transcriptomic analyses of matched tumors and nonneoplastic tissues and cancer cell lines, we reveal a comprehensive catalog of DoG RNA signatures. Through separate lines of evidence, we support the biological importance of DoG RNAs in carcinogenesis. First, we show tissue-specific and stage-specific differential expression of DoG RNAs in tumors versus paired normal tissues with their respective host genes involved in tumor-promoting versus tumor-suppressor pathways. Second, we identify that differential DoG RNA expression is associated with poor patient survival. Third, we identify that DoG RNA induction is a consequence of treating colon cancer cells with the topoisomerase I (TOP1) poison camptothecin and following TOP1 depletion. Our results underlie the significance of DoG RNAs and TOP1-dependent regulation of DoG RNAs in diversifying and modulating the cancer transcriptome.

## Teaser

DoG RNAs contribute to transcriptome diversity in human cancers.

## INTRODUCTION

Molecular discoveries revealing alterations in the cancer cell transcriptome have advanced our understanding of how cancer cells maintain their proliferative potential, evade tumor suppression and cell death, and undergo cancer cell invasion and metastasis (*1*). Long noncoding RNAs (lncRNAs) are emerging as key regulators of a variety of cellular processes that influence disease states including cancer (*2*). However, the common and specific lncRNA signatures across human cancers and the mechanisms driving alterations in lncRNA expression in carcinogenesis remain to be explored. An emerging class of lncRNAs referred to as downstream-of-gene (DoG)–containing transcripts (*3*–*5*) is initiated at the promoter of upstream protein-coding genes in response to stress stimuli that include viral infection (*6*, *7*), heat shock (*8*), and osmotic stress (*3*, *9*). Precisely, how stress signals trigger DoG RNA production is not fully understood. However, recent evidence has linked DoG biogenesis to defects in transcriptional termination (*9*, *10*). Moreover, while these lncRNAs are emerging as products of readthrough transcription in response to stress stimuli, their identity and classification in normal tissues (NTs) and tumorigenesis remain to be explored.

RNA polymerase II (RNAPII) is highly processive, and termination mechanisms ensure that RNAPII comes to a proper halt at protein-coding gene ends. Previous studies have established a model for coordination between pausing of the elongating RNAPII and recruitment of the 3′ end processing factors downstream of the polyadenylation sites on human genes (*11*, *12*). Recent studies provide support for integrator in targeting promoter-proximally paused RNAPII for termination that prevents elongation (*13*) and the induction of readthrough transcription (*9*, *10*). More recently, integrator has also been shown to support the elongation rate of RNAPII and render paused RNAPII into productive RNA synthesis (*14*). Specifically, hyperosmotic stress disrupts integrator associations with RNAPII, which decreases integrator binding to DNA and prompts enrichment of stress-induced DoG RNA production (*9*). Despite these advances, the factors and regulatory processes underlying alterations in RNAPII pause release frequency, termination, and DoG RNA production remain to be elucidated.

The essential enzyme, topoisomerase I (TOP1), supports RNAPII-dependent transcription through its contributions to preinitiation complex formation (PIC) (*15*–*21*) and transcriptional elongation (*22*). TOP1 through its recruitment by transcription factor IID (TFIID) has been shown to regulate PIC assembly through the formation of an active TFIID-TFIIA complex (*20*). TOP1 also directly acts to stimulate nucleosome disassembly and gene expression (*15*). In addition, paused promoters are particularly sensitive to the TOP1-selective inhibitor, camptothecin (CPT) (*23*), suggesting that TOP1 may regulate RNAPII pausing, a highly regulated step of the transcription cycle (*24*). Consistent with a role for TOP1 in pausing is a study revealing that bromodomain-containing protein 4 (BRD4) supports RNAPII pause release by enhancing TOP1 catalytic activity (*22*). While TOP1 is involved in the early steps of the RNAPII transcription cycle, less is known about TOP1’s role in regulating transcriptional termination other than TOP1 preventing replication stress at R loop–enriched transcription termination sites (*25*) and that coinhibition of TOP1 and BRD4 leads to the inhibition of transcription termination (*26*). Moreover, TOP1 overexpression has been identified to be a frequent event associated with colorectal cancer (CRC) (*27*, *28*). A first-line chemotherapy prolonging the survival of patient with CRC is the TOP1 inhibitor, CPT that acts by preventing TOP1 ligase activity, which leads to the persistence of single-stranded DNA breaks that evolve into double-stranded DNA breaks, and ultimately cell death (*29*–*31*). The impact of TOP1 on aberrant transcriptional control in CRC and the mechanisms underlying effective TOP1 targeting for cancer treatment largely remain to be elucidated.

Our study reveals distinct DoG RNA signatures in several major cancer types. We unveiled tumor- and stage-specific DoG RNAs that are produced from host genes functionally classified in contributing to tumor-promoting pathways. Comparatively, DoG RNAs produced in NTs and normal colon epithelial cells are produced from host genes involved in normal developmental and tumor-suppressor pathways. Moreover, the up-regulation and down-regulation of tumor-specific DoG RNAs across cancer stages are associated with an increased risk of mortality. We also reveal that TOP1-dependent dysregulation of DoG production has important implications in colon cancer. Specifically, TOP1 up-regulation in colon tumors is associated with decreased DoG RNA expression and conversely treating colon cancer cells with the anticancer TOP1 inhibitor CPT or TOP1 short hairpin RNA (shRNA) results in the induction of specific DoG RNAs. Our study provides an understanding of dysregulated DoG RNA signatures that have important implications for understanding several major cancer types and promise to elicit new therapeutic targets for tuning gene expression programs that shift disease-related networks.

## RESULTS

### Alterations in DoG RNA expression and length lead to transcriptomic imbalances with clinical relevance in major cancer types

**Fig. 1.**
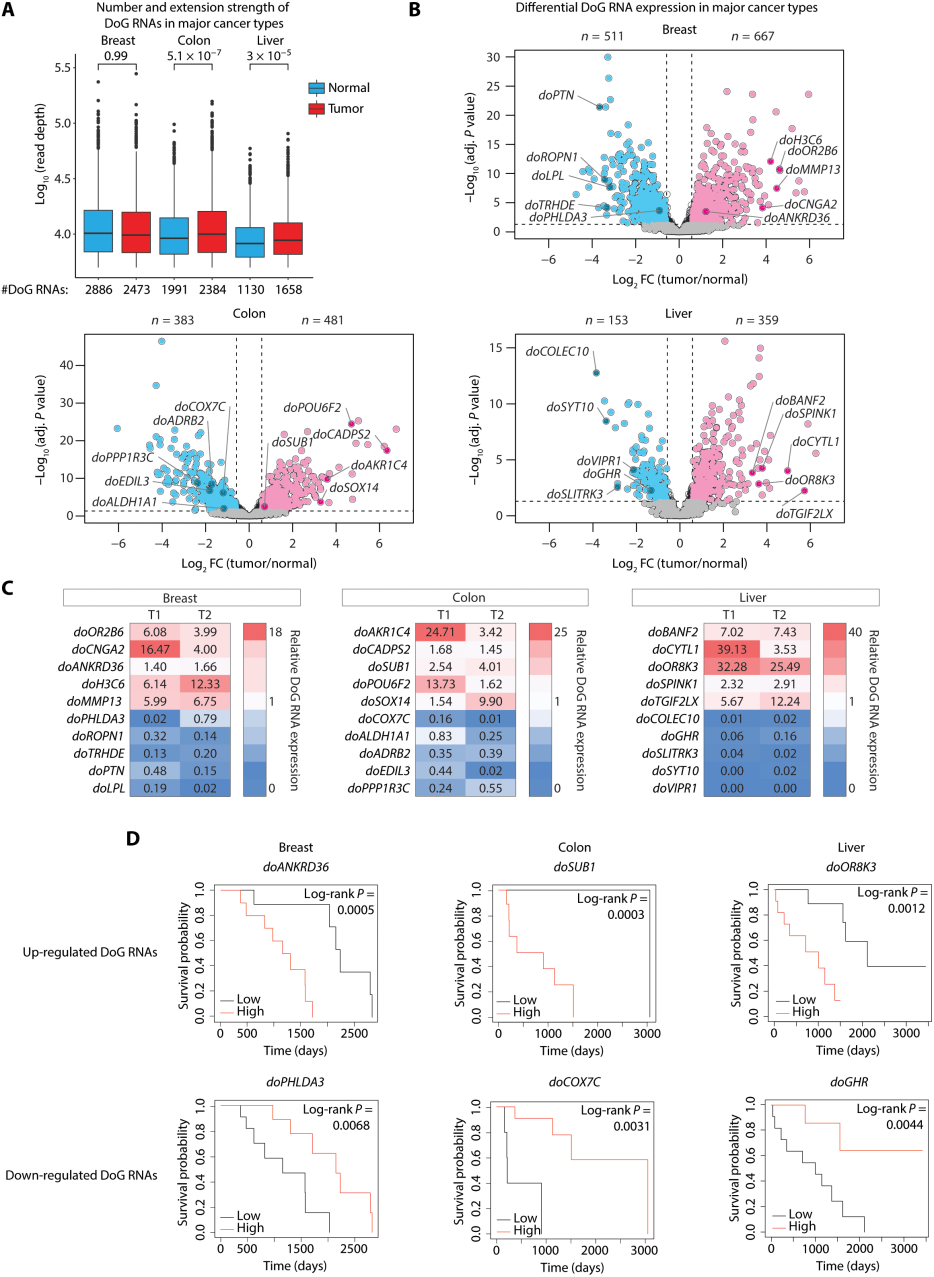
Readthrough transcription is prevalent in major cancers. (**A**) Number and extension strength of the DoG RNAs identified by DoGFinder (*33*) in breast, colon, and liver nonneoplastic (*n* = 22) and tumor (*n* = 22) samples from TCGA. The extension strength is shown in log_10_ scale. Box plots enclose values between first and third quartiles, midlines show medians, and whiskers extend to data points within 1.5 the interquartile range from the box; outliers are shown. Statistical significance was determined by one-way Wilcoxon rank sum test (alternative = “less”). *P* values include 0.99, 5.1 × 10^−7^, and 3 × 10^−5^ for NTs versus breast, colon, and liver tumors, respectively. (**B**) Volcano plots of the differentially expressed DoG RNAs (log_2_ FC > 0.58 or log_2_ FC < −0.58, *q* < 0.05) in breast, colon, and liver tumors compared with paired NTs. The significant down-regulated (blue) and up-regulated (pink) DoG RNAs are denoted, and the qPCR-validated DoG RNAs in (C) are denoted as darker shades of blue and pink. (**C**) qRT-PCR analysis of the denoted mRNAs and their respective DoG RNAs normalized to 18*S* ribosomal RNA (rRNA) in breast (left), colon (middle), and liver (right) tumors. Expression levels in tumors are relative to the levels in paired NTs. (**D**) Kaplan-Meier plots showing high versus low DoG RNA expression levels associated with overall patient survival in patients with breast (left), colon (middle), and liver (right) tumors. Statistical significance was determined using a log-rank test.

To identify and characterize DoG RNA transcriptional landscapes in three major cancers, we analyzed RNA sequencing (RNA-seq) data obtained from The Cancer Genome Atlas (TCGA) (*32*). Specifically, we analyzed RNA-seq data from a total of 44 patient tissues (22 paired tumors and NTs) from breast invasive carcinoma (BRCA), colon adenocarcinoma (COAD), and liver hepatocellular carcinoma (LIHC). DoG RNA calling was performed using DoGFinder (*33*) and the following criteria that included RNA-seq signal that shows >60% RNA-seq coverage and that is >5 kb of continuous read density downstream of the 3′ end of every annotated protein-coding gene locus. Thousands of DoG RNAs were identified in both paired NTs, breast (*n* = 2886), colon (*n* = 1991), and liver (*n* = 1130), and in the paired tumors, BRCA (*n* = 2473), COAD (*n* = 2384), and LIHC (*n* = 1658) (Fig. 1A and table S1). While a moderate decrease in DoG RNAs was identified in the paired NT and BRCA tumors (*n* = 2886 versus 2473), a moderate increase in DoG RNAs was identified in COAD (*n* = 1991 versus 2384) and LIHC (*n* = 1130 versus 1658) tumors (Fig. 1A). Another molecular feature that distinguished DoG RNAs in tumors versus paired NTs across COAD and LIHC tumors but not BRCA tumors was a significantly longer median extension strength or the continuous RNA-seq signal extending beyond the annotated gene end (Fig. 1A and table S1). Our definition of DoG RNA length or extension strength is dependent on the defined criteria for DoG calling (>60% RNA-seq coverage and >5-kb continuous read density). Thus, these measurements provide relative estimates rather than defined end points of DoG RNAs. The median extension strength was found to be significantly longer in the paired COAD (9.9 kb versus 9.1 kb) and LIHC (8.8 kb versus 8.3 kb) tumors versus paired NTs, respectively (Fig. 1A and table S1). In comparison, the extension strength was found to be comparable in the paired BRCA tumors and NTs (10 kb versus 9.7 kb), respectively (Fig. 1A and table S1).

Tumor-specific changes in DoG RNA expression levels were determined by measuring the log_2_-transformed ratio of the signal attributed to DoG transcripts produced from one host DoG-producing gene in a tumor relative to that same gene in a paired NT. DoG RNA expression levels are significantly altered (> or <1.5-fold, *q* < 0.05) in the transcriptomes of the tumors versus NTs for all cancer types. Compared to NTs, we identified a significant number of DoG RNAs that are either up-regulated (*n* = 667, 481, and 359, respectively) in BRCA, COAD, and LIHC or down-regulated (*n* = 511, 383, and 153, respectively) in BRCA, COAD, and LIHC tumors (Fig. 1B and table S1). Comparative analysis of the differentially regulated DoG RNAs in BRCA, COAD, and LIHC tumors revealed a small number of overlapping DoG RNAs (fig. S1, A and B). Specifically, only a few up-regulated (*n* = 71) and down-regulated (*n* = 16) DoG RNAs were found to overlap among the three tumor types (fig. S1, A and B). Thus, the vast majority of DoG RNAs that are up-regulated (*n* = 426, 256, and 178 in BRCA, COAD, and LIHC, respectively) and down-regulated (*n* = 371, 249, and 97 in BRCA, COAD, and LIHC, respectively) is tissue-specific (fig. S1, A and B). To further examine the relationship between differential mRNA and DoG RNA expression in tumors versus NTs, we determined the ratio of mRNAs with and without a DoG RNA extension in NTs versus BRCA, COAD, and LIHC tumors. First, we calculated the median transcript per million (TPM) values associated with the gene transcript versus over the DoG regions. We chose relative estimates of the DoG length based on the defined criteria for DoG calling (>60% RNA-seq coverage and >5-kb continuous read density) since the end points of DoG RNAs are not known. Notably, relative to paired NTs (read depth ratios of 0.5130 and 0.5837 in colon and liver tissues, respectively), transcription increases over the length of the DoG region in COAD and LIHC tumors as evidenced by the significant decreased ratio of read depth in COAD and LIHC tumors of 0.29 and 0.5148, respectively (fig. S1C). In comparison, transcription decreases over the DoG region in the BRCA tumors relative to paired NTs, which is evidenced by the significant increased read depth ratio in BRCA tumors versus NTs, of 0.3829 and 0.3592, respectively (fig. S1C). Thus, these data are consistent with significant increases in DoG RNA production in COAD and LIHC and significant decreases in DoG RNA production in BRCA exhibiting cancer relevancy.

Using quantitative polymerase chain reaction with reverse transcription (qRT-PCR), we confirmed differential expression of cancer-relevant DoG RNAs. For these analyses, we used RNA prepared from paired NT and BRCA, COAD, and LIHC tumors from two independent patients (Fig. 1C, T1 and T2). As shown in Fig. 1C, we confirmed five up-regulated and five down-regulated DoG RNAs that were among the top up-regulated and down-regulated DoG RNAs identified in the TCGA RNA-seq data for BRCA, COAD, and LIHC tumors (Fig. 1B and table S1). Specifically, as shown in Fig. 1C, relative to NTs, the significantly up-regulated DoG RNAs in BRCA tumors, T1 and T2, respectively, include *doOR2B6*, *doCNGA2*, *doANKRD36*, *doH3C6*, and *doMMP13*. In comparison, the significantly down-regulated DoG RNAs in BRCA tumors include *doPHLDA3*, *doROPN1*, *doTRHDE*, *doPTN*, and *doLPL*. In COAD tumors, the significantly up-regulated DoG RNAs include *doAKR1C4*, *doCADPS2*, *doSUB1*, *doPOU6F2*, and *doSOX14* and the significantly down-regulated DoG RNAs include *doCOX7C*, *doALDH1A1*, *doADRB2*, *doEDIL3*, and *doPPP1R3C* (Fig. 1C). In LIHC tumors, the significantly up-regulated DoG RNAs in T1 and T2 include *doBANF2*, *doCYTL1*, *doOR8K3*, *doSPINK1*, and *doTGIF2LX*, and the down-regulated DoG RNAs include *doCOLEC10*, *doGHR*, *doSLITRK3*, *doSYT10*, and *doVIPR1* (Fig. 1C). Functional pathway analysis of the DoG-producing host genes revealed that up-regulated DoG RNAs in BRCA, COAD, and LIHC tumors are associated with key regulators of tumor-promoting pathways, including G_2_-M checkpoint and glycolysis. These tumor-promoting pathways were shared among all three of the tumor types including BRCA, COAD, and LIHC (fig. S1D, top). Other tumor-promoting pathways identified for BRCA, COAD, or LIHC tumors included E2F targets, mammalian target of rapamycin complex 1 signaling, angiogenesis, and epithelial mesenchymal transition (fig. S1D, top). In comparison, functional pathway analysis of the DoG-producing host genes associated with down-regulated DoG RNAs revealed tumor-suppressor pathways [p53 pathway and ultraviolet (UV) response] or normal cellular pathways including cholesterol homeostasis, heme metabolism, xenobiotic metabolism, bile acid metabolism, and coagulation (fig. S1D, bottom). The high-level significance and functional coherence of functional pathways associated with the up-regulated DoG-producing host genes suggest that the differential expression of DoG RNAs exhibits cancer relevancy.

To investigate whether the dysregulation of DoG RNA expression across tumors is associated with overall survival (OS), we next integrated the DoG RNA signatures in BRCA, COAD, and LIHC tumors with patient survival information from 22 patients using the TCGA data (table S1). As illustrated by the Kaplan-Meier survival plots (Fig. 1D), both up-regulation and down-regulation of DoG RNAs in BRCA, COAD, and LIHC tumors are associated with poor patient prognosis and lower survival probabilities. Specifically, patients with BRCA with high versus low expression levels of *doANKRD36* (1142 days versus 1620 days, respectively) and low versus high expression levels of *doPHLDA3* (811 days versus 1620 days, respectively) had a shorter median OS (Fig. 1D, left). Patients with COAD with high versus low expression levels of *doSUB1* (291 versus 887 days, respectively) and low versus high expression levels of *doCOX7C* (210 versus 926 days, respectively) have a shorter median OS (Fig. 1D, middle). Patients with LIHC with high versus low expression levels of *doOR8K3* (662 versus 1560 days, respectively) and low versus high expression levels of *doGHR* (1005 versus 1363 days, respectively) were found to have a shorter median OS (Fig. 1D, right).

To further examine the cancer relevancy of the DoG RNAs identified in Fig. 1B, we also considered alterations in DoG RNA expression relative to tumor stage. Relative to the DoG RNAs identified in stage 1 tumors, we identified differentially expressed DoG RNAs in late-stage (stages 2 and 3) breast, colon, and liver tumors (fig. S1E and table S2). In BRCA tumors, a higher number of differentially expressed DoG RNAs were identified in stage 3 versus stage 2 that are down-regulated (*n* = 17 versus 8, respectively) and up-regulated (*n* = 19 versus 10, respectively) (fig. S1E and table S2). Specifically, among the down-regulated DoG RNAs in BRCA tumors, 4 DoG RNAs are specific to stage 2 and include *doCCR7*, *doFAM186A*, *doTAL2*, and *doADAMTS15*, and 13 are specific to stage 3 and include *doSIRPG*, *doAPOBEC3D*, *doSPIC*, *doTASL*, *doCLEC4C*, *doTRAT1*, *doFGD2*, *doSELPLG*, *doTNIP3*, *doIL7R*, *doTAP2*, *doRLN2*, and *doIL12RB1* (table S2). Among the up-regulated DoG RNAs in BRCA tumors, we identified 8 DoG RNAs that are specific to stage 2 and include *doC1orf116*, *doWDR43*, *doMELK*, *doZWINT*, *doSEC24B*, *doAKAP1*, *doNCAPG2*, and *doAZIN1* versus 17 DoG RNAs that are specific to stage 3 and include *doSUDS3*, *doNEUROD2*, *doDNAJB12*, *doZNF251*, *doBARX1*, *doKITLG*, *doRRM2*, *doZNF706*, *doEPHA3*, *doTKT*, *doSLC25A32*, *doSNX13*, *doASCL2*, *doGSDMB*, *doNUDCD1*, *doCFAP58*, and *doESF1* (table S2). In comparison, the differentially expressed DoG RNAs in COAD and LIHC tumors were lower in stage 3 versus stage 2 (fig. S1E). Specifically, we identified (5 versus 12) down-regulated and (4 versus 10) up-regulated DoG RNAs in stage 3 versus stage 2 COAD tumors, respectively (fig. S1E and table S2). In LIHC stage 3 versus stage 2 tumors, we identified (*n* = 5 versus 21) down-regulated and (*n* = 4 versus 5) up-regulated DoG RNAs, respectively (fig. S1E and table S2). Specifically, among the down-regulated DoG RNAs in COAD tumors, we identified 11 stage 2–specific DoG RNAs that include *doCTPS2*, *doNEBL*, *doHNRNPA0*, *doFZD6*, *doGATM*, *doRRAGB*, *doUBQLN2*, *doFIBIN*, *doMFSD14B*, *doCPSF6*, and *doITGB1BP2* (table S2). Among the up-regulated DoG RNAs in COAD tumors, we identified seven stage 2–specific DoG RNAs that include *doNFAM1*, *doTTLL6*, *doRSPH1*, *doHTR1D*, *doTBC1D16*, *doPIK3R2*, and *doMEFV* and only one stage 3–specific DoG RNA, *doTMEM200A* (fig. S1E and table S2). Among the down-regulated DoG RNAs in LIHC tumors, we identified 19 stage 2–specific DoG RNAs that include *doSAA2*, *doFBXL4*, *doZNF648*, *doRTP3*, *doMOGAT1*, *doADRB2*, *doNMD3*, *doMBL2*, *doDHTKD1*, *doCCL16*, *doNUPR1*, *doGREM2*, *doZNF563*, *doPAQR9*, *doSLC22A25*, *doCYP2J2*, *doADRA1A*, *doMAT1A*, and *doTMEM176A* and 3 stage 3–specific DoG RNAs that include *doPRAMEF10*, *doCCDC177*, and *CYP2G1P* (fig. S1E and table S2). Among the up-regulated DoG RNAs in LIHC tumors, we identified 5 stage 2–specific DoG RNAs, *doGP2*, *doPSPH*, *doIL1RAPL1*, *doASXL2*, and *doHCN4* and 4 stage 3–specific DoG RNAs, *doH3C8*, *doGLUL*, *doRGSL1*, and *doSLC37A1* (fig. S1E and table S2).

Using patient survival information from the 22 patients in TCGA further revealed that differential expression of the stage-specific DoG RNAs is associated with patient OS. As illustrated by the Kaplan-Meier survival plots (fig. S1F), the up-regulation of late-stage (stage 3–specific) DoG RNAs, *doZNF251* and *doGSDMB*, in BRCA tumors was associated with poor patient survival with a median survival of 1247 versus 1286 days and 1142 versus 1611 days, respectively. In addition, down-regulated stage 2–specific DoG RNA, *doITGB1BP2*, and up-regulated stage 2–specific DoG RNA, *doFZD6*, are also associated with poor patient prognosis and lower survival probabilities in patients with COAD cancer with median survival of 214 versus 906 days and 214 versus 1026 days, respectively (fig. S1F). Together, the differential, tissue-specific, and stage-specific DoG RNA expression in tumorigenesis is consistent with DoG RNAs serving as a key source of transcriptome diversity that have clinical and biological significance in major cancers.

### DoG RNAs reshape the transcriptome of human colon tumors

**Fig. 2.**
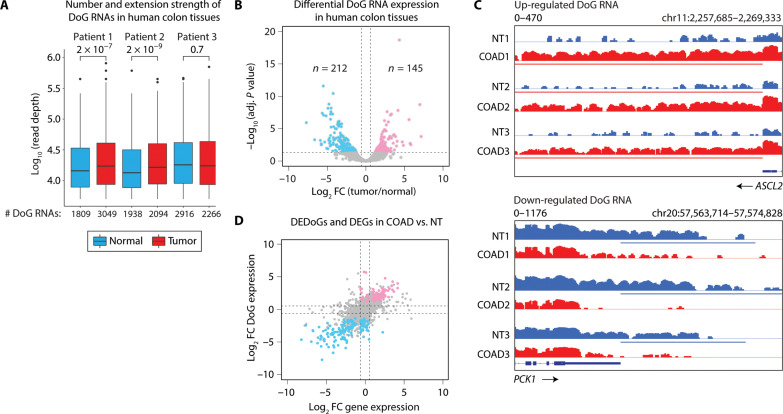
DoG RNAs in colorectal carcinoma tissues are differentially expressed and associated with tumorigenic pathways. (**A**) Number and extension strength of DoG RNAs identified by DoGFinder (*33*) in three nonneoplastic (NT, blue) and three COAD (red) tumors. The extension strength is shown in log_10_ scale. Box plots enclose values between first and third quartiles, midlines show medians, and whiskers extend to data points within 1.5 the interquartile range from the box; outliers are shown. Statistical significance was determined by one-way Wilcoxon rank sum test (alternative = less). *P* values include 2 × 10^−7^, 2 × 10^−9^, and 0.7 for patients 1 to 3, respectively. (**B**) Volcano plot showing differentially expressed DoG RNAs (log_2_ FC > 0.58 or log_2_ FC < −0.58, *q* < 0.05) in the same three NT and three COAD tissues shown in (A). The significantly down-regulated (blue) and up-regulated (pink) DoG RNAs are shown. (**C**) Integrative Genomics Viewer (IGV) tracks of total RNA-seq signal in log reads per kilobase of transcript per million mapped reads (RPKM) at *ASCL2* and *PCK1* loci in three paired NT and COAD samples shown in (A) and (B). NT and COAD tissues are represented in blue and red, respectively. The horizontal bars define the DoG region determined by DoGFinder (*33*). (**D**) Scatter plot showing log_2_ FC expression for the DoG-producing genes on the *x* axis and the log_2_ FC DoG RNA expression on the *y* axis. Down-regulated and up-regulated DoG RNAs (log_2_ FC > 0.58 or log_2_ FC < −0.58, *q* < 0.05) and their host genes’ expression (silenced: log_2_ FC < −0.58 and *q* < 0.05; not changed: −0.58 ≤ log_2_ FC ≤ 0.58 and *q* ≥ 0.05; activated: log_2_ FC > 0.58 and *q* < 0.05) in COAD samples relative to NT are represented in blue and red, respectively. DEDoGs, differentially expressed DoGs.

To avoid potential ambiguities associated with differences in experimental execution and analysis of the published datasets, we performed nascent RNA-seq using ribosomal RNA (rRNA)–depleted RNA (total RNA-seq) to identify and confirm differential DoG RNA expression in three paired COADs and NTs. Thousands of DoG RNAs were identified in the NTs and COADs from each of the patients (Fig. 2A). Relative to matched NTs, a higher DoG number was identified in two of the three COADs (*n* = 1809 versus 3049 and 1938 versus 2094, respectively), while a moderate decrease in DoG RNA number was identified in the third COAD (*n* = 2916 versus 2266, respectively) (Fig. 2A and table S3). Consistent with the TCGA data analysis showing significantly longer DoG RNA extension strengths in colon tumors versus paired NTs (Fig. 1A), the DoG RNA extensions were significantly longer in the two COADs with higher DoG number relative to the NTs (Fig. 2A). Specifically, the longer median extension lengths in the COADs consisted of 17.2 and 16.4 kb versus 14.4 and 13.4 kb in the NTs, respectively (Fig. 2A and table S3). Comparison of the DoG RNAs identified in the 3 COADs and 22 COADs from TCGA revealed overlap of 1710 DoG-producing host genes (fig. S2A, left). Specifically, 72% of the DoG-producing host genes in TCGA COADs overlap with the host genes identified in the three COADs, and, conversely, 88% of the host genes in the three COADs overlap with those identified in the TCGA datasets (fig. S2A, left). Similarly, we identified an overlap of 1529 DoG-producing host genes in the three paired colon NTs and TCGA colon NTs (fig. S2A, right). We identified a similar percentage (70%) of host genes in the TCGA NTs that overlap with the host genes in the three NTs as were identified for the overlapping DoG-producing genes in the TCGA and three COADs (72%) (fig. S2A, right). Similarly, we identified a comparable percentage (77%) of the host genes in the three NTs that overlap with those in the TCGA datasets (fig. S2A, right).

We next performed differential expression analysis for the DoG RNAs in the three paired NTs versus COADs, which revealed significantly (> or <1.5-fold, *q* < 0.05) down-regulated (*n* = 212, 5%) and up-regulated (*n* = 145, 3%) DoG RNAs in the tumors (Fig. 2B and table S3). Differential DoG RNA expression in COADs versus NTs is further evidenced by the increased and decreased RNA-seq signal mapping downstream of the transcription end site (TES) of protein-coding genes (fig. S2B). Specifically, we found that the *ASCL2* mRNA is associated with a DoG RNA in all three COADs versus the paired NTs (Fig. 2C). In comparison, the *PCK1* mRNA is associated with a DoG RNA specifically in the three NTs, but not paired COADs (Fig. 2C).

We next wanted to determine whether the expression of DoG RNAs relates to the expression levels of their host genes. Log_2_ fold change (FC) in the expression of DoG RNAs versus DoG-producing host genes in the three COADs versus paired NTs revealed that a large number (*n* = 57, 39%) of up-regulated DoG RNAs are produced from transcriptionally up-regulated DoG-producing genes (Fig. 2D and table S4). In comparison, a smaller number of up-regulated DoG RNAs are produced from DoG-producing genes whose expression levels are not changing (*n* = 17, 12%) or are down-regulated (*n* = 0, 0%) in COAD tumors relative to NTs (Fig. 2D and table S4). Moreover, down-regulated DoG RNAs in COAD tumors relative to NTs are largely produced from DoG-producing genes (*n* = 110, 52%) that are also down-regulated in COAD tumors (Fig. 2D and table S4). A smaller number of down-regulated DoG RNAs were associated with host genes whose expression levels are not changing (*n* = 47, 22%) or are up-regulated (*n* = 1, 0.5%) in COADs relative to NTs (Fig. 2D and table S4). These data draw a strong parallel between the transcription levels of DoG RNAs and their respective host genes. Together, our RNA-seq and qRT-PCR analyses reveal congruence between the NT and cancer-relevant DoG RNAs identified in the TCGA data and these independent RNA-seq analyses of colon tissues.

### DoG RNAs lead to a transcriptomic imbalance in colon cancer cell lines

**Fig. 3.**
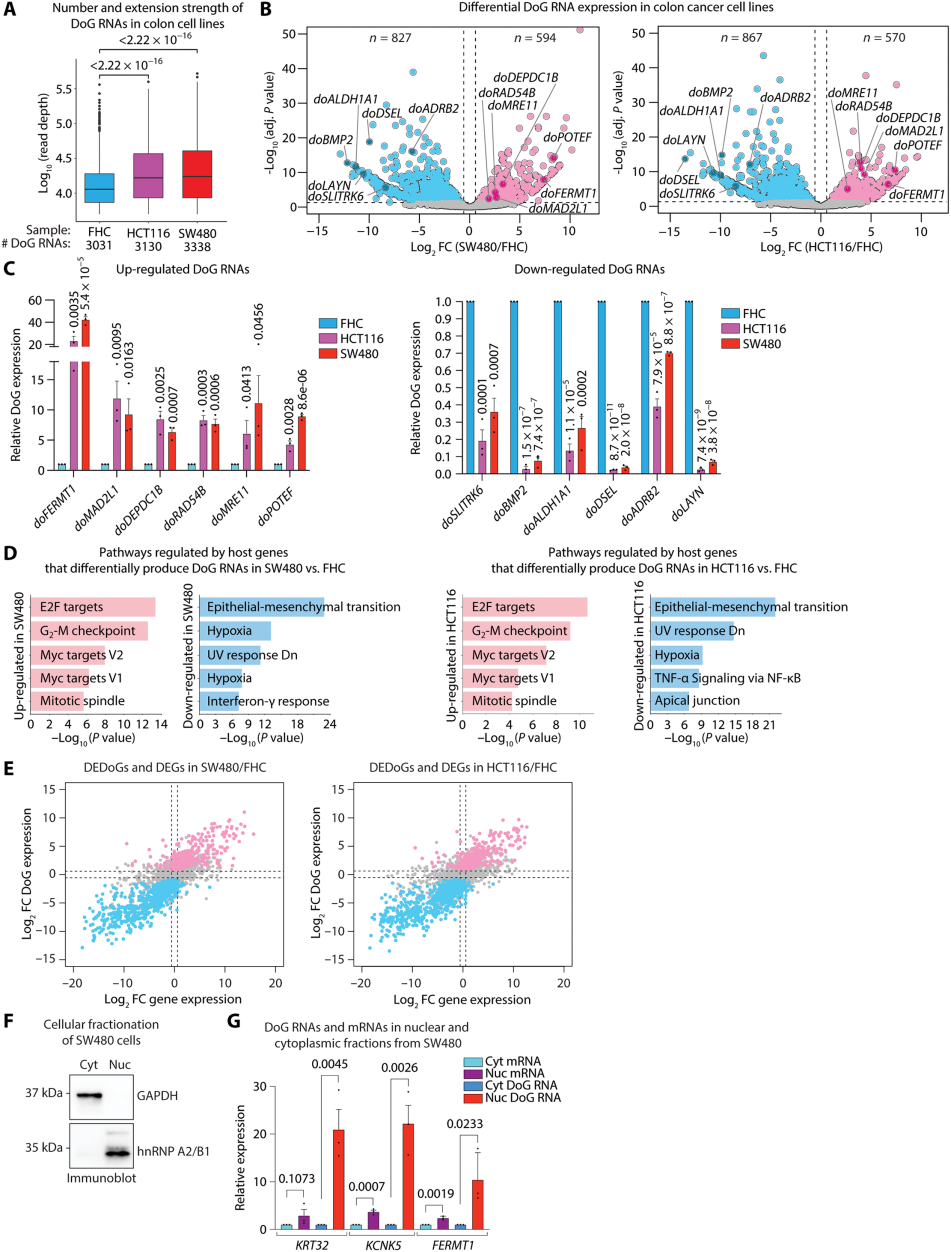
DoG RNA production is prevalent in colorectal carcinoma cell lines. (**A**) DoG number and extension strength identified in FHC, HCT116, and SW480 cells by DoGFinder (*33*). Box plots enclose values between first and third quartiles, midlines show medians, and whiskers extend to data points within 1.5 the interquartile range from the box; outliers are shown. Statistical significance determined by one-way Wilcoxon rank sum test (alternative = less). *P* < 0.05. Extension strength is shown in log_10_ scale. (**B**) Volcano plot of differentially expressed DoG RNAs (log_2_ FC > 0.58 or log_2_ FC < −0.58, *q* <0.05) in SW480 versus FHC and HCT116 versus FHC cells. (**C**) qRT-PCR analysis of DoG RNAs denoted in (B) and normalized with GAPDH. Expression levels in SW480 or HCT116 cells are relative to the levels in FHC cells. Data represent the mean and SEM that are representative of three independent experiments. *P* values are shown. (**D**) Top five (*P* < 0.05) Molecular Signatures Database (MSigDB) pathways for host genes showing up-regulated and down-regulated DoG RNAs in SW480 versus FHC (left) and HCT116 versus FHC (right). TNF-α, tumor necrosis factor–α; NF-κB, nuclear factor κB. (**E**) Scatter plots showing log_2_ FC for the DoG-producing gene on the *x* axis and log_2_ FC for the DoG RNA expression on the *y* axis. Down-regulated and up-regulated DoG RNAs (log_2_ FC > 0.58 or log_2_ FC < −0.58, *q* < 0.05) and their host genes’ expression (silenced: log_2_ FC < −0.58 and *q* < 0.05; not changed: −0.58 ≤ log_2_ FC ≤ 0.58 and *q* ≥ 0.05; activated: log_2_ FC > 0.58 and *q* < 0.05) in SW480 versus FHC (left) and HCT116 versus FHC (right) cells. (**F**) Immunoblot of GAPDH and heterogeneous nuclear ribonucleoprotein (hnRNP) A2/B1 from SW480 cytoplasmic and nuclear extracts. Image is representative of three independent experiments. (**G**) qRT-PCR analysis of the denoted mRNAs and their respective DoG RNAs normalized with GAPDH using RNA from SW480 cytoplasmic and nuclear fractions. Data represent the mean and SEM that are representative of three independent experiments. *P* values are shown.

To refine our observations that there exist differentially expressed DoG RNAs with an extended length in tumors versus paired NTs, we used total RNA-seq to identify DoG RNAs in colon cancer cell lines HCT116 and SW480 versus normal colon epithelial fetal human colon (FHC) cells. A significant number of DoG RNAs (*n* = 3031, 3130, and 3338) exhibiting an average extension strength (11.4, 16.6, and 17.3 kb) were identified in FHC, HCT116, and SW480 cells, respectively (Fig. 3A and table S5). By comparing differential expression analysis of DoG RNAs in SW480 and HCT116 versus colon FHC cells, we identified a comparable number of significantly up-regulated [*n* = 594 (18%) or *n* = 570 (18%)] and down-regulated [*n* = 827 (25%) or *n* = 867 (28%)] DoG RNAs in SW480 and HCT116 cells, respectively (Fig. 3B and table S5). Specifically, heatmaps (fig. S3A) and specific gene examples, *TTK* and *EXT1* (fig. S3B), reveal the up-regulated versus down-regulated DoG RNAs past the annotated gene ends in HCT116 and SW480 versus FHC cells. To confirm DoG RNA identification in colon cancer SW480 cells, we performed genome-wide mapping of active RNAPII using precision nuclear run-on sequencing (PRO-seq) (*34*), which revealed comparable identification of DoG RNAs as observed in our RNA-seq data (fig. S3C). We also confirmed by qRT-PCR analyses that differentially expressed DoG RNAs were found to be significantly up-regulated and down-regulated in the COAD tumors (Fig. 1B and table S1) and the cancer cell lines SW480 and HCT116 (Fig. 3B). As shown in Fig. 3C, relative to FHC cells, the significantly up-regulated DoG RNAs in SW480 and HCT116 cells respectively include *doFERMT1* (42- and 23-fold), *doMAD2L1* (9.3- and 11.9-fold), *doDEPDC1B* (6.4- and 8.5-fold), *doRAD54B* (7.7- and 8.3-fold), *doMRE11* (11- and 6-fold), and *doPOTEF* (8.9- and 4.3-fold). In comparison, the significantly down-regulated DoG RNAs in SW480 and HCT116 cells respectively include *doSLITRK6* (2.8- and 5-fold), *doBMP2* (13- and 36-fold), *doALDH1A1* (3.7- and 7.4-fold), *doDSEL* (27- and 44-fold), *doADRB2* (1.4- and 2.6-fold), and *doLAYN* (14- and 40-fold) (Fig. 3C).

Consistent with the identified congruence between DoG RNAs identified in COADs and colon cancer cell lines, we observed similar functional pathways associated with the DoG-producing host genes in HCT116 and SW480 cells (Fig. 3D) and DoG-producing genes in COAD tumors (fig. S1D). Specifically, we identified that the host genes associated with up-regulated DoG RNAs are linked to protumorigenic pathways including G_2_-M checkpoint, E2F targets and MYC targets (Fig. 3D). In comparison, the host genes associated with down-regulated DoG RNA production in HCT116 and SW480 revealed functional pathways that include the epithelial mesenchymal transition, UV response down, and hypoxia (Fig. 3D). In addition, consistent with our analysis of DoG RNA signatures in COAD tumors (Fig. 2D), we found that the expression levels of DoG RNAs in SW480 and HCT116 cells are largely consistent with the expression levels of their corresponding DoG-producing host gene (Fig. 3E and table S6). Specifically, log_2_ FC in the expression of DoG RNAs versus DoG-producing mRNAs in both SW480 and HCT116 cells versus FHC cells revealed that the vast majority (*n* = 433 and 373, 73% and 65%, respectively) of DoG RNAs is induced from transcriptionally active DoG-producing genes (Fig. 3E and table S6). In comparison, a smaller number of DoG RNAs are induced from DoG-producing genes whose expression is not changing (*n* = 66 and 77, 11% and 14%, respectively) or that become silenced (*n* = 24 and 22, 4% and 4%, respectively) in the cancer cells (Fig. 3E and table S6). Moreover, a large number (*n* = 694 and 735, 84% and 85%, respectively) of down-regulated DoG RNAs are associated with DoG-producing genes that are also down-regulated in SW480 and HCT116 cells (Fig. 3E and table S6). A smaller number of down-regulated DoG RNAs were associated with host genes that are not changing (*n* = 71 and 74, 9% and 8%, respectively) or that are up-regulated (*n* = 13 and 8, 2% and 1%, respectively) in SW480 and HCT116 cells (Fig. 3E and table S6).

We next investigated the cellular localization of DoG RNAs by isolating RNA from cytoplasmic and nuclear fractions prepared from SW480 cells. The subcellular fractions were confirmed by immunoblot analysis of glyceraldehyde-3-phosphate dehydrogenase (GAPDH) and heterogeneous nuclear ribonucleoprotein (hnRNP) A2/B1 as cytoplasmic and nuclear markers, respectively (Fig. 3F). qRT-PCR analysis of the fractionated RNA was performed, and the nuclear expression levels of DoG RNAs and their associating mRNAs were normalized to their respective expression levels in cytoplasmic fractions. Notably, we found that the DoG RNAs, *doKRT32* (21-fold), *doKCNK5* (22.2-fold), and *doFERMT1* (10.4-fold), are significantly enriched in the nuclear relative to the cytoplasmic fraction (Fig. 3G). In comparison, the DoG-associated mRNAs, *KRT32* (2.9-fold), *KCNK5* (3.7-fold), and *FERMT1* (2.4-fold), were also enriched but to a lesser extent than the DoG RNAs in the nuclear fractions, which is consistent with the higher-level enrichment of the DoG-associated mRNAs versus DoG RNAs in the cytoplasmic fractions (Fig. 3G). Overall, these findings support the nuclear enrichment of mRNAs with DoG RNA extensions.

### DoG RNA production in colon cancer cells is regulated by TOP1 inhibition and depletion

**Fig. 4.**
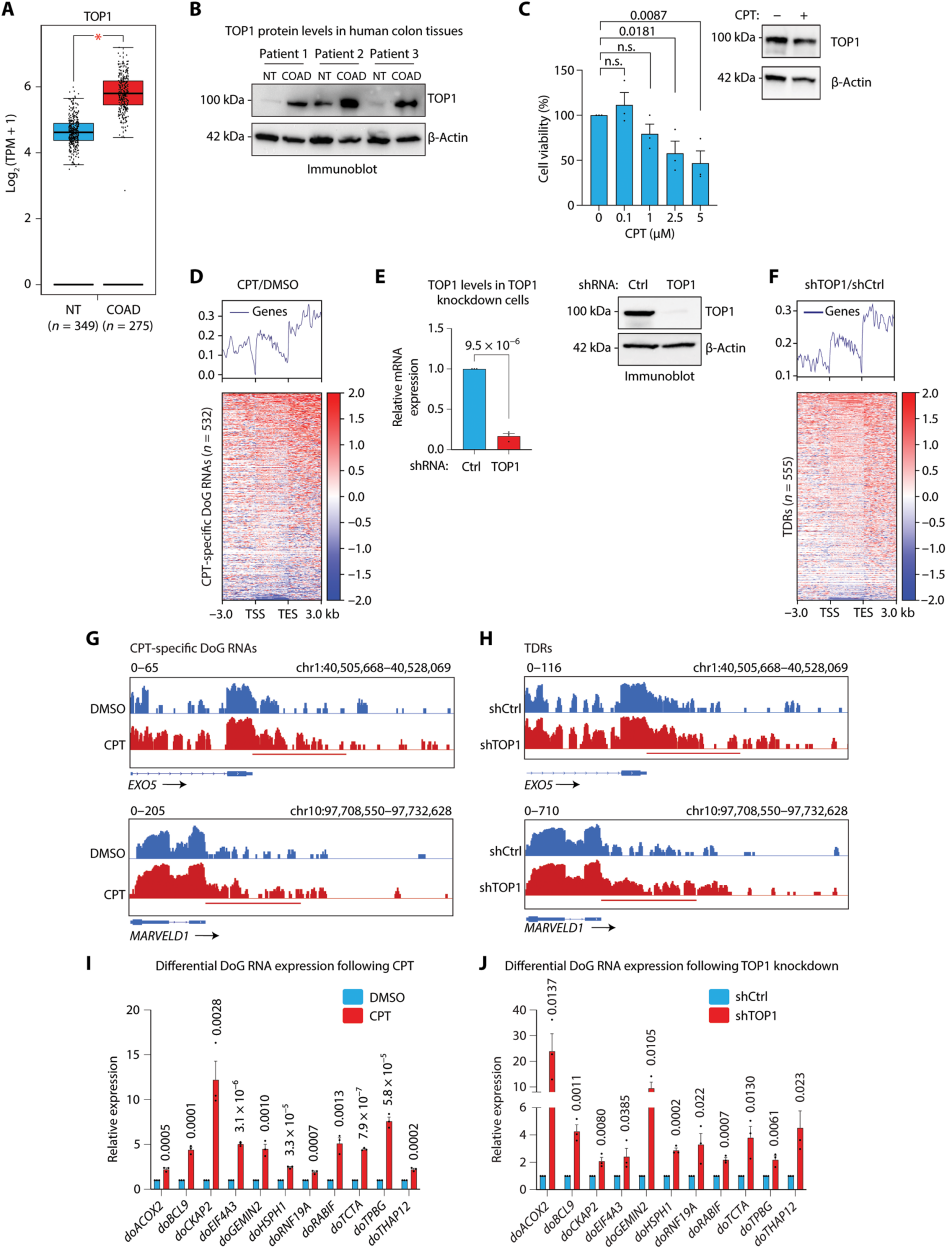
TOP1 regulates DoG RNA production in colorectal carcinoma. (**A**) Box with jitter plot for *TOP1* RNA-seq levels in 349 NTs and 275 COADs determined with GTEx and TCGA data from GEPIA server (*32*, *35*, *66*). Statistical significance was determined by one-way analysis of variance (ANOVA) test (*P < 0.05). (**B**) Immunoblot analysis of TOP1 and β-actin from paired NT and COADs from three patients. (**C**) Left: Dose response (0, 0.1, 1, 2.5, and 5 μM) for CPT treatment in SW480 cells. SW480 cell number was examined relative to CPT treatment for 24 hours. Right: Immunoblot analysis of TOP1 and β-actin in SW480 treated with 1.15 μM CPT or equal volume DMSO for 3 hours. *P* values are shown. n.s., nonsignificant. (**D**) Heatmap of RNA-seq distribution spanning 3-kb upstream of TSS to 3-kb downstream of TES of the CPT-specific DoG RNAs (*n* = 532). The log_2_ ratio of RPKM is represented in SW480 cells treated with CPT versus DMSO. (**E**) qRT-PCR (left) and immunoblot analysis (right) of SW480 cells stably expressing control (Ctrl) or TOP1 shRNA. Data represent the mean and SEM representative of three independent experiments. *P* = 9.5 × 10^−6^. (**F**) Heatmap of RNA-seq distribution spanning 3-kb upstream of the TSS to 3-kb downstream of the TES of the TDR genes (*n* = 555). The log_2_ ratio of RPKM is represented in TOP1 knockdown versus shCtrl SW480 cells. IGV tracks of RNA-seq signal (RPKM) at the *EXO5* and *MARVELD1* loci in SW480 cells treated with (**G**) DMSO versus CPT or (**H**) expressing Ctrl versus TOP1 shRNA. The horizontal bar defines the DoG region determined by DoGFinder (*33*). qRT-PCR analysis of DoG RNA induction in SW480 cells treated with (**I**) CPT versus DMSO or (**J**) shTOP1 versus shCtrl. Data represent the mean and SEM that are representative of three independent experiments. *P* values are shown.

Having identified that DoG RNAs are differentially expressed and exhibit clinical significance in colon cancer, we next examined whether the Food and Drug Administration–approved TOP1 inhibitor, CPT, alters DoG RNA expression. Consistent with CPT being an effective therapeutic in treating advanced colon cancer in the clinic is the finding that *TOP1* is significantly up-regulated in COAD tumors and high *TOP1* expression is associated with high CPT sensitivity (*29*, *30*). Using RNA-seq data from 275 COADs versus 349 NTs from TCGA and the Genotype-Tissue Expression (4GTEx) databases (*32*, *35*), we identified significantly higher *TOP1* mRNA levels in COAD tumors (Fig. 4A). Similarly, as shown in Fig. 4B, immunoblot analysis revealed higher TOP1 protein levels in the three COADs relative to the matched NTs that were used for DoG RNA identification in Fig. 2A. We further extended the importance of *TOP1* up-regulation to other cancers by examining *TOP1* mRNA levels in the same 44 patient tissues (22 paired NTs and tumors) from breast, colon, and liver tissues that were used for DoG RNA calling (Fig. 1, A and B) from TCGA (*32*). Consistent with up-regulated *TOP1* RNA expression in COADs (Fig. 4A), *TOP1* expression is also significantly (*P* = 2.82 × 10^−8^) up-regulated in the additional COAD tumors relative to NTs (fig. S4A). Moreover, significant *TOP1* up-regulation was also identified in BRCA versus NTs (*P* = 0.001468) but not LIHC tumors (fig. S4A). To examine whether *TOP1* up-regulation in COAD is associated with changes in DoG production, we next parsed RNA-seq data from the 22 COADs used from TCGA (Fig. 1A) into two subsets with either high (*n* = 11) or low (*n* = 11) *TOP1* mRNA levels (fig. S4B). Notably, COADs with high TOP1 levels are associated with significantly fewer (*n* = 1319) DoG RNAs relative to COADs with low TOP1 levels (*n* = 1901) (fig. S4B). This finding is consistent with an anticorrelation between TOP1 and DoG RNA levels.

Having found that colon cancer–associated *TOP1* up-regulation is correlated with lower DoG RNAs levels, we hypothesized that the therapeutic benefits of inhibiting TOP1 activity with CPT may be linked to increased DoG RNA levels in colon cancer. To examine this possibility, we determined the 25% inhibitory concentration (IC_25_) of CPT in SW480 cells. Specifically, we examined the effects of various CPT concentrations (0, 0.1, 1, 2.5, and 5 μM) for 24 hours on the proliferation of SW480 cells. These data revealed that 1.15 μM CPT resulted in a 75% cell survival (Fig. 4C, left). We performed follow-up RNA-seq experiments in SW480 cells using the determined IC_25_ to maintain high levels of cell viability and lessen the potential side effects from CPT treatment. Total RNA-seq was performed to identify alterations in DoG RNA levels following 3-hour CPT treatment in which TOP1 levels were not affected by CPT (Fig. 4C, right). Relative to dimethyl sulfoxide (DMSO), we identified 532 protein-coding genes that specifically produce DoG RNAs following CPT treatment (fig. S4C and table S7). Notably, CPT treatment results in elevated DoG production as evidenced by the increase in transcription signal past the annotated TESs of these 532 protein-coding genes (Fig. 4D). Consistent with CPT primarily affecting DoG RNA versus mRNA expression levels of these 532 DoG-producing genes is the minimal change in RNA-seq signal spanning from the transcriptional start site (TSS) to the TES of these DoG-producing genes following CPT treatment (Fig. 4D). These findings suggest that CPT releases a potent inhibition of DoG RNA production at a subset of genes in colon cancer cells.

To examine a direct role for TOP1 in the inhibition of DoG RNA production, we next performed RNA-seq in SW480 colon cancer cells expressing shRNAs against TOP1. As shown in Fig. 4E, relative to a nontargeting shRNA against LacZ [control (Ctrl)], TOP1 shRNA markedly reduced *TOP1* mRNA (left) and TOP1 protein (right) levels. Notably, we identified a comparable number of DoG-producing genes (*n* = 555) that are specific to TOP1 versus the Ctrl knockdown (fig. S4D and table S7) as were identified following CPT treatment (*n* = 532) (fig. S4C and table S7). For brevity purposes, we refer to the DoG RNAs that are specific to TOP1 knockdown as TOP1 DoG RNAs (TDRs). Consistent with TOP1 primarily regulating DoG RNA versus TDR gene expression is the minimal change in RNA-seq signal spanning from the TSS to the TES (Fig. 4F). Further analysis of qRT-PCR confirmed a significant increase in the expression levels of DoG RNAs, *doDAPK3* (3-fold), *doBRF1* (3.9-fold), and *doMAPK9* (2.6-fold), following TOP1 shRNA–mediated knockdown in SW480 cells (fig. S4F). Moreover, TOP1 regulation of DoG RNAs, *doDAPK3*, *doBRF1*, and *doMAPK9*, was further confirmed following TOP1 depletion using small interfering RNAs (siRNAs) directed against different regions of TOP1 mRNA relative to the TOP1 targeted shRNA oligos (fig. S4G) and following TOP1 knockdown in a second colon cancer HCT116 cell line (fig. S4H).

Comparative analysis of the DoG RNA signatures specifically produced following CPT treatment (Fig. 4D and fig. S4C), and TOP1 knockdown (Fig. 4F and fig. S4D) revealed 38 (~7%) overlapping DoG RNAs (fig. S4E). Using the hypergeometric test, we revealed that the number of overlapping DoG-producing genes achieves a statistically significant level within the total of 20,046 protein-coding genes (*P* = 1.04 × 10^−7^). Nonetheless, the DoG RNAs specific to CPT (*n* = 494, 93%) and TOP1 knockdown (*n* = 517, 93%) suggest that TOP1 regulation of DoG RNAs is not only dependent on its catalytic activity but may also relate to other unknown TOP1 functions. The identification of overlapping DoG RNAs is evidenced by the increase in RNA-seq signal past the TESs of the *EXO5* and *MARVELD1* gene loci that are among the DoG-producing genes showing induced DoG RNA expression following CPT treatment (Fig. 4G) and TOP1 knockdown (Fig. 4H). In addition, qRT-PCR analyses confirmed potent induction of DoG RNAs following CPT treatment (Fig. 4I) and TOP1 knockdown (Fig. 4J). Specifically, following CPT treatment, a significant induction was observed for *doACOX2* (2.2-fold), *doBCL9* (4.4-fold), *doCKAP2* (12.2-fold), *doEIF4A3* (5.1-fold), *doGEMIN2* (4.5-fold), *doHSPH1* (2.5-fold), *doRNF19A* (1.9-fold), *doRABIF* (5.1-fold), *doTCTA* (4.5-fold), *doTPBG* (7.6-fold), and *doTHAP12* (2.2-fold) (Fig. 4I). We further revealed that TOP1 knockdown results in a significant and comparable fold induction of these same DoG RNAs. Specifically, following TOP1 knockdown, we observed a significant induction of *doACOX2* (24-fold), *doBCL9* (4.3-fold), *doCKAP2* (2.1-fold), *doEIF4A3* (2.4-fold), *doGEMIN2* (9.6-fold), *doHSPH1* (2.9-fold), *doRNF19A* (3.3-fold), *doRABIF* (2.2-fold), *doTCTA* (3.8-fold), *doTPBG* (2.2-fold), and *doTHAP12* (4.5-fold) (Fig. 4J). Together, these findings underscore the importance of relieving the TOP1-dependent block of DoG production in colon cancer, which is overcome by CPT and TOP1 knockdown and therefore may have therapeutic implications in colon cancer.

### Paused RNAPII and catalytically inactive TOP1 accumulate at TES-proximal regions

**Fig. 5.**
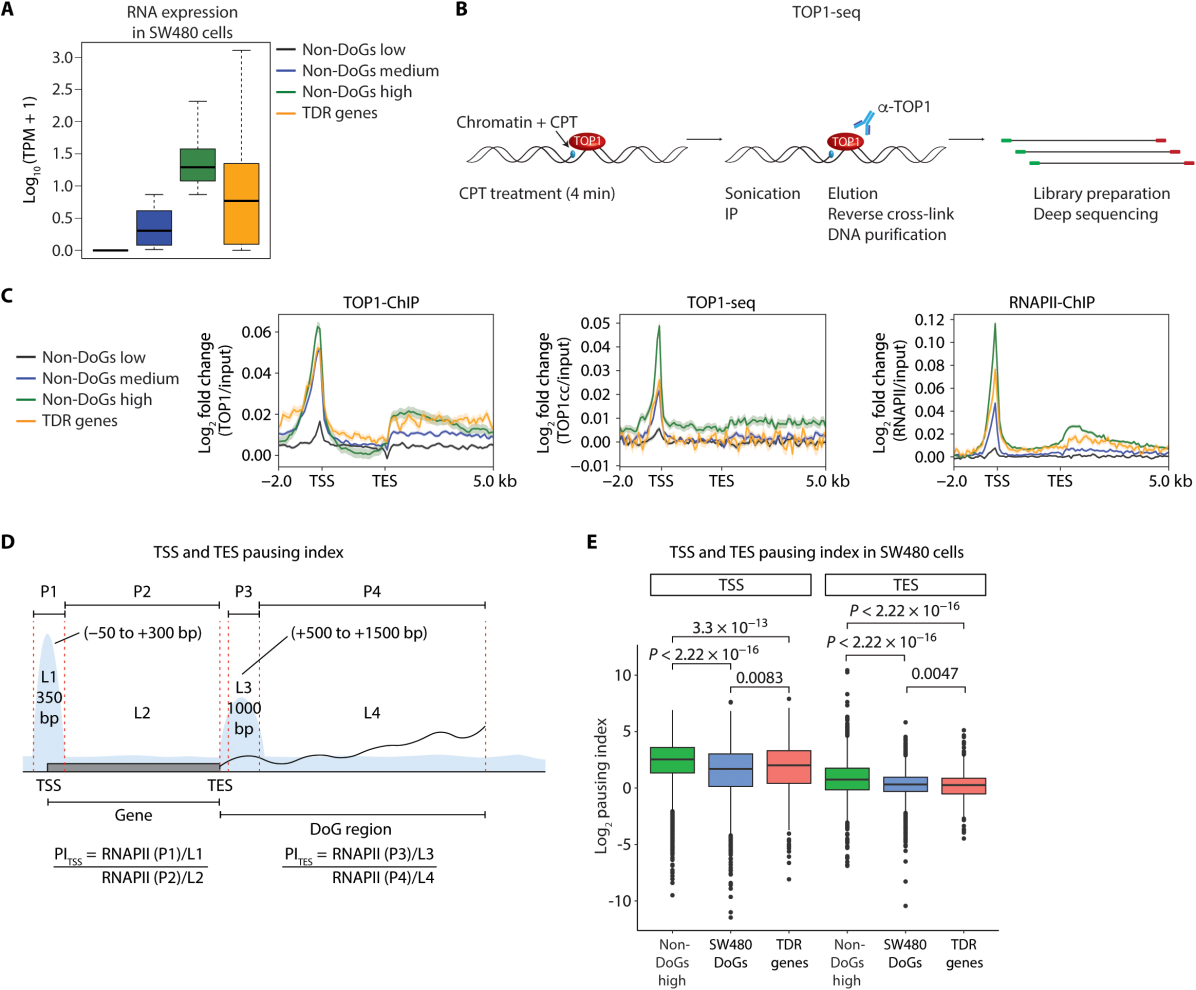
RNAPII and TOP1 accumulate at the promoter- and TES-proximal regions of paused genes. (**A**) Box plot showing the counts of TDRs and non-DoG–producing genes with low, medium, and high transcriptions in SW480 cells. The counts were grouped using the bottom and top quartiles into three groups, low (quartile 1), medium (quartile 2), and high (quartile 3) expressions according to the total RNA-seq reads. Box plots enclose values between first and third quartiles, midlines show medians, and whiskers extend to data points within 1.5 the interquartile range from the box; outliers are shown. (**B**) Schematic of TOP1-seq, a method to identify TOP1cc (*22*). (**C**) Metaplots of TOP1, TOP1-seq, and RNAPII ChIP-seq signal at TDRs (*n* = 555) and non-DoG–producing genes with low, medium, and high transcriptions in SW480 cells. TOP1-seq and ChIP-seq signal is represented as log_2_-transformed FC of bins per million over input and spans 2-kb upstream of the TSS to 5-kb downstream of the TES. (**D**) Illustration depicting the pause index determination for TDR genes. The TSS pause index is defined as the ratio of PRO-seq reads at paused site 1 at the TSS (P1, spanning from −50 to +300 bp) over the PRO-seq reads at gene body (P2, spanning from +300 to end of the gene). The TES pause index is defined as the ratio of PRO-seq reads at paused site 2 on TES (P3, spanning from +500 to +1500 bp) over the PRO-seq reads at DoG region identified by DoGFinder (*33*) (P4, spanning from +1500 to end of the DoG defined by DoGFinder) (*33*). (**E**) Box plot showing the TSS and TES pause indices in highly expressed non-DoG–producing genes (green), SW480 DoG-producing genes (blue), and TDR genes (red) in SW480 cells. Statistical significance was determined by two-way Wilcoxon rank sum test.

To investigate the mechanisms underlying TOP1’s role in preventing DoG RNA production in colon cancer, we monitored the global relationship between TOP1 catalytic activity and TOP1 and RNAPII binding profiles at TDR host genes and non-DoG–producing genes that, similar to TDR genes, are transcribed at high levels (Fig. 5A). Using SW480 colon cancer cells, we performed chromatin immunoprecipitation followed by deep sequencing (ChIP-seq) for RNAPII and TOP1 and measured TOP1 catalytic activity using TOP1 sequencing (TOP1-seq; Fig. 5B), a method for identifying only catalytically engaged TOP1 (TOP1cc) (*22*). To examine the relationship between TOP1cc, TOP1, and RNAPII profiles and transcription, we next parsed the TDR host genes and non-DoG–producing genes in SW480 cells using the lower and upper quartiles into three subsets, low (quartile 1), medium (quartile 2), and high (quartile 3) transcription levels as measured by RNA-seq (Fig. 5A). The TDR host genes are transcribed at high levels that are comparable to the transcription levels of the highly transcribed non-DoG–producing genes (Fig. 5A). High levels of TOP1, RNAPII, and TOP1cc were identified at the TSSs of TDR and mediumly and highly transcribed non-DoG–producing genes (Fig. 5C). While the TOP1, RNAPII, and TOP1cc enrichment levels were significantly lower at the low transcribed genes (Fig. 5C). The increased enrichment of TOP1, RNAPII, and TOP1cc at the TSSs is consistent with increased supercoiling that coincides with the significantly higher transcription levels observed at TDR and mediumly to highly transcribed non-DoG–producing genes. Specifically, TOP1 enrichment at the TSSs of TDRs and mediumly and highly non-DoG–producing genes was found to be comparable and significantly higher relative to the low transcribed non-DoG–producing genes (Fig. 5C). Notably and despite comparable levels of TOP1 binding, TOP1cc enrichment at the TDR and mediumly transcribed genes was comparable but lower than TOP1cc accumulation at highly transcribed non-DoG–producing genes (Fig. 5C). Similarly, RNAPII enrichment was comparable to TOP1cc profiles with lower levels of RNAPII binding at the TDR and mediumly transcribed non-DoG–producing genes relative to the highly transcribed non-DoG–producing genes (Fig. 5C). At the TES-proximal regions, a second peak of TOP1 and RNAPII binding was observed that, similar to the TSS peak, was higher at the TDR host genes and the highly transcribed versus the lowly and mediumly transcribed non-DoG–producing genes (Fig. 5C). Despite high levels of TOP1 and RNAPII binding at the TDR TESs, we observed negligible levels of TOP1cc at the TES-proximal regions of TDRs and lowly and mediumly transcribed non-DoG–producing genes (Fig. 5C). In comparison, TOP1cc levels at the highly transcribed non-DoG–producing genes remained high over the gene bodies and past the TESs (Fig. 5C). Together, our data suggest a role for TOP1 and TOP1cc at TDR TSSs and, conversely, a role for TOP1 at TDR TESs that is likely independent of its catalytic function.

The high-density RNAPII binding that overlaps with TOP1 binding at the TSS and TES of TDR host genes and highly transcribed non-DoG–producing genes is consistent with RNAPII pausing patterns. To examine whether the TDR host genes are largely occupied by paused RNAPII, we used our PRO-seq data in SW480 cells to calculate the pausing index (PI) for all DoG-producing genes versus two subsets of non-DoG–producing genes, the TDR host genes (yellow; Fig. 5A) and the highly transcribed non-DoG–producing genes (green; Fig. 5A). PRO-seq enables detection of nascent transcripts at a single-nucleotide resolution that are specifically produced by paused or elongating polymerases versus stalled or arrested polymerases. As shown in Fig. 5D, we calculated the PI at the TSS (PI_TSS_) by measuring the ratio of RNAPII density at the TSS [L1, −50 to +300 base pairs (bp)] relative to the RNAPII density in the gene body (L2, +300 bp to the annotated end of the genes). In addition, we measured the PI associated with 3′ gene ends (PI_TES_) by dividing the normalized RNAPII density at the TES-proximal region (L3, +500 to +1500 bp) by the RNAPII density downstream of the TES (L4, +1500 bp to +10,000 bp). At the TSSs, a PI_TSS_ greater than 2 was identified, which is consistent with a strong promoter bias of paused RNAPII at all three subsets of genes (Fig. 5E). Moreover, we found that the median PI_TSS_ was significantly higher for highly transcribed non-DoG–producing genes (*n* = 5294) as compared to the DoG-producing genes (*n* = 3462) and TDR host genes (*n* = 555) (Fig. 5E). Examination of the PI_TES_ further revealed enrichment of paused RNAPII, which is albeit lower than the PI_TSS_ observed at all three gene subsets (Fig. 5E). Together, these data are consistent with all three subsets of genes consisting of paused RNAPII at the TSS and TES. Notably and consistent with DoG production, we found that the median PI_TES_ associated with DoG-producing genes is significantly lower than the median PI_TES_ for the highly transcribed non-DoG–producing genes. Notably, the median PI_TES_ for the TDR host genes was also found to be significantly lower than that of the non-DoG–producing genes and the DoG-producing genes (Fig. 5E). Consistent with the higher median PI_TES_ observed at the non-DoG–producing and DoG-producing genes relative to TDRs is a higher average profile of PRO-seq density past the TESs of these two subsets of genes (fig. S5). In addition, consistent with the TDRs having the lowest median PI_TES_ is the finding that the PRO-seq signal density remains at a comparable and lower level at the TES-proximal region and beyond (~10-kb downstream) relative to the non-DoG–producing genes and DoG-producing genes (fig. S5). Together, these data demonstrate that TDR genes exhibit high levels of concordance between the binding profiles of TOP1 and paused RNAPII at the promoter- and TES-proximal regions. Moreover, differences in the localization and accumulation of TOP1 binding versus TOP1cc accumulation suggest that TOP1 is likely to exhibit other, noncatalytic roles at TES-proximal regions.

### TOP1 is essential for RNAPII termination at hundreds of TDR host genes

**Fig. 6.**
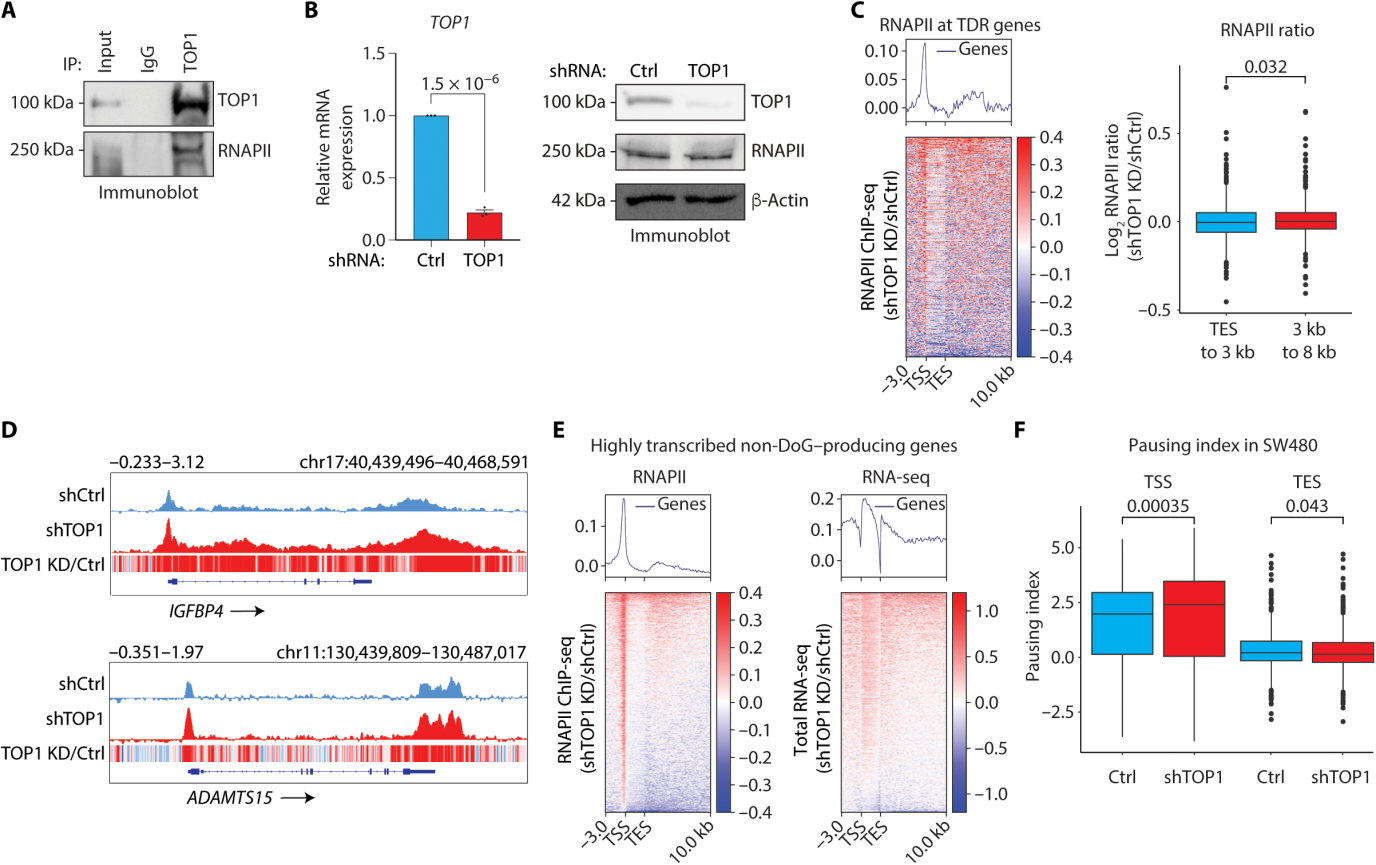
TOP1 associates with RNAPII to regulate TDR genes. (**A**) Coimmunoprecipitation (IP) with TOP1 and immunoglobulin G (IgG) antibodies from SW480 nuclear extracts and immunoblot analysis of RNAPII and TOP1. An image is shown that is representative of three independent experiments. (**B**) Left: qRT-PCR analysis of *TOP1* mRNA. Data represent the mean and SEM of three independent qRT-PCR experiments. *P* = 1.5 × 10^−6^. Right: Immunoblot analysis of TOP1, RNAPII, and β-actin in SW480 cells expressing Ctrl and TOP1 shRNA. A representative image is shown that is representative of three independent experiments. (**C**) Heatmap of RNAPII ChIP-seq distribution spanning 3-kb upstream of the TSS to 10-kb downstream of the TES of the TDR genes (*n* = 555). The RNAPII ChIP-seq represents the log_2_ ratio of ChIP-seq signal in TOP1 knockdown (KD) over shCtrl in SW480 cells. Box plot showing the pause index at TDR genes at two regions, TES to 3 kb and 3 to 8 kb past the TES in SW480 cells expressing shTOP1 over shCtrl. Statistical significance was determined by two-way Wilcoxon rank sum test. *P* = 0.032. (**D**) IGV tracks of RNAPII ChIP-seq signal at the *IGFBP4* and *ADAMTS15* loci in SW480 cells expressing Ctrl and TOP1 shRNA. (**E**) Heatmaps of RNAPII ChIP-seq and RNA-seq distribution spanning 3-kb upstream of the TSS to 10-kb downstream of the TES of the highly transcribed non-DoG genes. The log_2_ ratio of RNA-seq signal (RPKM) is represented in shTOP1 versus shCtrl SW480 cells. The RNAPII ChIP-seq signal is represented as the log_2_ ratio of ChIP-seq signal in shTOP1 over shCtrl SW480 cells. (**F**) Box plot showing the TSS and TES pause indices (calculated from RNAPII ChIP-seq) on TDRs genes in SW480 cells expressing Ctrl and TOP1 shRNA. Statistical significance was determined by two-way Wilcoxon rank sum test. *P* = 0.043 and *P* = 0.00035.

Given the notable overlap of TOP1 and RNAPII binding profiles, we next examined whether TOP1-dependent regulation of DoG RNA expression is associated with alterations in RNAPII behavior. First, we examined whether TOP1 and RNAPII form functional associations in SW480 cells. As shown in Fig. 6A, an antibody specifically recognizing TOP1 coimmunoprecipitated RNAPII from nuclear extracts prepared from SW480 cells. These results establish physiological associations between TOP1 and RNAPII that are consistent with the global overlap in their binding profiles (Fig. 5C).

Having identified enrichment of TOP1 and RNAPII binding at the TSS- and TES-proximal regions of highly transcribed TDR genes, we next examined whether TOP1 is directly regulating DoG RNA production by modulating RNAPII chromatin engagement. To examine this possibility, we performed RNAPII ChIP-seq following TOP1 shRNA-mediated knockdown, which significantly decreased *TOP1* mRNA and protein levels without affecting RNAPII protein levels (Fig. 6B). Inspection of the heatmap analyses (Fig. 6C, left) and individual TDR host genes (Fig. 6D) revealed increased RNAPII binding at the promoter-proximal regions and past TESs under TOP1 versus Ctrl knockdown conditions. Consistent with defects in RNAPII pause release at promoter-proximal regions is the finding that TOP1 depletion resulted in increased RNAPII at the promoter region but only moderate/negligible increases in RNAPII binding were observed spanning the region from the TSS to the TES (Figs. 5C, left, and 6D). Notably, at TDR genes where an increased DoG RNA-seq signal is observed following TOP1 knockdown (Fig. 4F), we also identify increased RNAPII binding downstream of the TES-proximal region [Fig. 6, C (left) and D], which is consistent with defects in transcriptional termination following TOP1 knockdown. Specifically, quantitative analysis of RNAPII binding at the TES-proximal region (TES to 3-kb downstream) and downstream of the TES-proximal region (3 to 8 kb) revealed that RNAPII binding is significantly increased past the TES-proximal regions (Fig. 6C, right). Moreover, at highly transcribed non-DoG–producing genes, negligible changes in RNAPII binding were observed over the DoG regions following TOP1 depletion (Fig. 6E, left), which is consistent with these genes not producing DoG RNAs (Fig. 6E, right). However, a notable increase in RNAPII binding was observed at the promoter-proximal regions of the non-DoG–producing genes following TOP1 knockdown (Fig. 6E, left). This increase in promoter-proximal RNAPII binding is consistent with RNAPII pause release defects, which is common to both highly transcribed DoG-producing (Fig. 6C) and non-DoG–producing genes (Fig. 6E, left). The high levels of promoter-proximal RNAPII following TOP1 knockdown are not accompanied by changes in RNAPII binding within the coding regions of the non-DoG–producing (Fig. 6E, left) genes. Moreover, these nominal changes in RNAPII binding are associated with only a moderate increase in RNA-seq signal over the gene bodies (Fig. 6E, right).

To further investigate the defects caused by TOP1 loss on aberrant RNAPII pause release and RNAPII accumulation at TDR promoter-proximal regions and downstream of the TES-proximal regions, we calculated the PI_TES_ and PI_TSS_ using the RNAPII ChIP-seq data in Ctrl versus TOP1 knockdown. Consistent with our PI calculations using PRO-seq data in SW480 cells, which revealed 331 TDR genes with paused RNAPII (PI_TSS_ > 2) (Fig. 5E), the RNAPII ChIP-seq data in shCtrl cells revealed a comparable number of 336 TDR genes with paused RNAPII (PI_TSS_ > 2) (Fig. 6F). Notably, relative to the shCtrl, TOP1 knockdown significantly altered both PI_TSS_ and PI_TES_ of TDR genes (Fig. 6F). Specifically, a significant increase in the PI_TSS_ was observed, which is consistent with the significant increase in RNAPII accumulation at the TDR promoter-proximal regions following TOP1 knockdown (Fig. 6F, left). Notably, however, a significantly lower PI_TES_ at TDR genes was observed in TOP1 knockdown versus Ctrl cells (Fig. 6F, right). The significant decrease in the PI_TES_ (Fig. 6F, right) is consistent with the observed increases in RNAPII levels that are detected downstream of the TES-proximal region of the TDR genes following TOP1 knockdown (Fig. 6C). Collectively, these data reveal different roles for TOP1 in regulating RNAPII binding at the TSS and TES of TDR genes. Specifically, our data support a role for TOP1 in promoting the release of promoter-proximal paused RNAPII and in establishing efficient transcription termination by RNAPII at TDR host genes.

## DISCUSSION

**Fig. 7.**
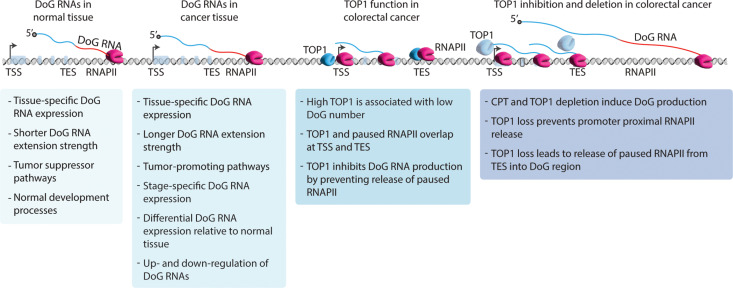
Working model for how DoG RNAs reshape normal and cancer transcriptomes. Comparative analyses of DoG RNA signatures in normal versus cancer tissues provide previously unknown insights into this emerging class of differentially expressed tissue- and stage-specific ncRNAs. Dysregulated expression of DoG RNAs in breast, liver, and colon tumors is significantly correlated with poor patient survival. Up-regulated DoG RNAs are associated with DoG-producing host genes that exhibit tumor-promoting functions, and down-regulated DoG RNAs are linked to host genes involved in normal developmental and tumor-suppressor pathways. Treatment of colon cancer cells with the TOP1 poison, CPT, leads to an induction of DoG RNA production, which is consistent with DoG RNAs exhibiting a potential therapeutic benefit in patients with colon cancer. Mechanistically, we confirm that TOP1 depletion promotes DoG RNA induction by lowering the RNAPII PI at the TES and promoting RNAPII release well beyond the ends of TDR host genes.

While ncRNAs make up the vast majority of our transcriptome, our understanding of the noncoding genome remains limited. Among the emerging classes of ncRNAs are DoG RNAs that are induced in response to stress stimuli including osmotic stress (*3*, *9*), viral infection (*6*, *7*), and heat shock (*8*). Several unsolved questions regarding DoG RNAs exist. For example, are DoG RNAs produced in normal cellular contexts and in human disease states including cancer? Can differential expression analyses of DoG RNAs in normal versus cancer tissues provide new glimpses into the biological differences between a healthy versus cancer cell? What makes certain protein-coding genes in the genome prone to DoG RNA production, and, relatedly, are there specific transcriptional regulators that Ctrl DoG RNA expression? In our study, we address these questions through global identification and classification of differentially expressed DoG RNA signatures in paired normal and cancer tissues. Our data support a model (Fig. 7) for DoG RNAs in diversifying both nonneoplastic and cancer cell transcriptomes. These new insights stem from unveiling alterations in DoG RNA expression that are tissue- and cancer stage–specific, and the up-regulation and down-regulation of DoG RNAs are associated with decreased OS of patients with breast, liver, and colon cancer (Fig. 7). Moreover, our finding that alterations in DoG RNA production in colon cancer are likely to have clinical relevance is further supported by our data revealing that DoG RNAs are induced in colon cancer in response to chemotherapeutic TOP1 poison CPT and TOP1 depletion (Fig. 7).

To reach a deeper understanding of alterations in the cancer cell transcriptome, we sought to demonstrate the existence and cancer specificity of DoG RNAs. By using an approach using TCGA RNA-seq data from matched tumors and NTs and an established pipeline, DoGFinder (*33*), we reveal a comprehensive catalog of previously unrecognized DoG RNA signatures that reshape both normal and cancer transcriptomes. Among the molecular features of DoG RNAs, we found that differences in tumors versus paired NTs are changes in DoG number and extension strength. Specifically, moderate increases in DoG number and significantly longer DoG RNA extension strength were identified in COAD and LIHC tumors, and conversely deceased DoG number and nonsignificant changes in DoG extension strength were identified in BRCA tumors. Since DoG RNAs are continuous ncRNA extensions of their upstream protein-coding genes, our study suggests the significance of longer mRNAs with DoG RNA extensions in tumorigenesis. Consistent with aberrant DoG-producing mRNAs exhibiting cancer relevancy is our finding of a significantly lower stochiometric ratio of correctly transcribed mRNAs relative to mRNAs with DoG RNA extensions in paired COAD and LIHC tumors compared to their paired NTs. Conversely, we identified a higher ratio of correctly transcribed mRNAs relative to those with DoG RNAs in BRCA tumors, suggesting that the loss versus gain of DoG RNAs may be most relevant for breast tumorigenesis. Recent studies examining shifts in gene length support a paradigm for age-associated imbalances in gene length (*36*). In addition to aging, length-associated transcriptomic imbalances have also been linked to Alzheimer’s disease (*37*). While various length transcripts are enriched under specific biological contexts, future work is needed to determine causality of the various length-associated transcripts including DoG RNAs in both normal development and disease states.

Cancer progression involves multiple genetic and epigenetic events that involve both gain-of-function oncogenes and loss-of-function tumor suppressors. Comparative analysis of DoG RNA signatures in paired NTs versus tumors of breast, colon, and liver origins revealed both a gain and loss of DoG RNA expression. Consistent with dysregulated DoG RNA expression underlying tumorigenesis is the finding that DoG-producing host genes with up-regulated DoGs encode for transcripts involved in tumor-promoting functional pathways including G_2_-M checkpoint, glycolysis, and E2F targets. Comparatively, functional annotations observed for DoG-producing genes with down-regulated DoG RNAs revealed enrichment of normal cellular and developmental processes and tumor-suppressor pathways. Consistent with the gain and loss of DoG RNAs overlapping with the biological hallmarks of tumorigenesis and tumor suppression, respectively, is the finding that both the up-regulation and down-regulation of DoG RNAs significantly correlated with poor patient survival. Moreover, we revealed that DoG RNAs largely exhibit tissue-specific expression patterns, which is similar to other classes of lncRNAs. In addition, similar to other lncRNAs is the finding that DoG RNAs are expressed in a stage-specific manner. Alterations in the expression of late stage-specific DoG RNAs in BRCA and COAD tumors, respectively, are linked to a significant reduction in patient OS. Future investigations are needed to discern regulatory factors and mechanisms underlying the differential regulation of this emerging class of ncRNAs in normal developmental and disease states including cancer.

The tissue- and stage-specific expression of DoG RNAs is consistent with these molecules serving as attractive therapeutic targets that inform prognosis and treatment of patients with colon cancer. Our study suggests that triggering DoG induction may exude a therapeutic benefit in treating patients with colon cancer. This is evidenced by the enhanced levels of DoG production in colon cancer cells that are observed following inhibition of TOP1 catalytic activity with CPT. Consistent with the clinical benefit of enhancing DoG production with CPT is our finding that TOP1 up-regulation, which is common in COAD tumors, is associated with lower DoG number. Thus, the therapeutic advantage of CPT that occurs in tumors with up-regulated TOP1 may be linked, at least in part with CPT-dependent enhanced levels of DoG RNA production. However, additional mechanistic insight is needed to fully understand how differential TOP1 levels are related to changes in DoG RNA production in human cancers. For example, our findings revealed that high TOP1 levels are associated with increased DoG production in COADs when compared to paired NTs. However, our stratification of COAD tumors into low versus high TOP1 levels revealed high versus low DoG numbers, respectively. This suggests that there are likely various mechanisms underlying the roles of TOP1 in contributing to alterations in DoG production in human cancers.

Consistent with a direct role for TOP1 in regulating DoG production is our finding that TOP1 depletion, similar to TOP1 inhibition with CPT, results in genes that escape transcriptional termination and support high levels of DoG production in colon cancer cells. How precisely enhanced DoG production yields a clinical benefit for patients with cancer remains unclear as the functions of DoG RNAs also largely remain a mystery. One possibility is that DoG RNAs induced by CPT and TOP1 loss will induce transcriptional stress and lead to cell cycle arrest and cell death. Recent evidence supports a role for transcription inhibition in leading to increased DoG production (*38*). Our study connects up-regulated DoG RNAs in colon cancer to host mRNAs that are known to regulate cell cycle arrest including G_2_-M phase arrest. CPT is also known to induce G_2_-M phase arrest mediated by reactive oxygen species (*39*), which, together with previous studies showing that oxidative stress inhibits 3′ end cleavage of nascent transcripts and leads to readthrough transcription (*9*), suggests a mechanism by which CPT-induced DoG RNAs could lead to cell cycle arrest. Another possibility is that TOP1-regulated DoG RNAs will induce R loop formation, replication stress, and cellular senescence, which has been previously shown for RNAPII transcription elongation factor, SPT6 whose loss leads to the formation of DoG RNAs that are prone to R loop formation (*40*). Consistent with this model is the known role that TOP1 plays in preventing replication stress at R loop–enriched transcriptional termination sites (*25*). The increased levels of DoG production that we observe following TOP1 loss and inhibition will overlap with DNA replication origins, which, in turn, can alter replisome progression and lead to replication stress. Moreover, an increased likelihood of a collision arising between replication and transcription machinery would be expected on the basis of increases in DoG extension strength. This possibility is therefore more likely in COAD and LIHC tumor relative to their paired NTs where we observed increased DoG extension strength and following CPT and TOP1 knockdown that enhanced DoG expression. While our study supports a role for TOP1 loss and inhibition in promoting DoG production, additional investigations are warranted to discern whether TOP1 also regulates down-regulation of DoG RNAs. Our study reveals that both down-regulation and up-regulation of DoG RNAs in human cancers are prevalent and associated with poor prognosis. Thus, it will be important going forward to uncover other regulatory factors and mechanisms underlying both up-regulation and down-regulation of DoG production in normal development and disease.

TOP1 has long been viewed as a key determinant of gene Ctrl that is governed by its regulation of the PIC (*15*–*21*) and RNAPII elongation (*22*). Our study further supports a role for TOP1 in facilitating RNAPII elongation by preventing aberrant accumulation of RNAPII at promoter-proximal regions. This role for TOP1 in RNAPII pause release is evidenced by the increased enrichment of paused RNAPII at the promoter region of TDR genes that we observed following TOP1 knockdown. The increased accumulation of paused RNAPII is likely related to the known role of BRD4 in facilitating RNAPII pause release by stimulating TOP1 activity via phosphorylation of the RNAPII C-terminal domain (CTD) (*22*). According to the twin domain model (*41*), as DNA moves through the active site of RNAPII, positive supercoils are generated ahead, and negative supercoils trail behind RNAPII. If the supercoiling tension is not removed by TOP1, then this would oppose RNAPII pause release and therefore lead to the buildup of RNAPII that we observe at the promoter region. The requirement for stimulating TOP1 activity particularly at the promoter-proximal regions of TDR genes would be expected on the basis of our finding that there exist high levels of TOP1cc at this subset of genes, which is consistent with the high levels of TDR expression that would be expected to generate high levels of torsional strain.

Our studies further unveil that TOP1 is also a key regulator of gene Ctrl at the level of termination. Notably, the identification of increased DoG production, together with negligible changes in the expression of the DoG-producing gene following TOP1 depletion, is consistent with our data showing that TOP1 loss raises the TSS PI but lowers the TES PI. These findings contribute to a growing area of interest that includes defining the regulatory mechanisms underlying termination that is the least understood step of the RNAPII transcription cycle. The mechanisms underlying these defects in transcriptional termination following TOP1 loss remain unclear. First, note that we observed particularly high levels of TOP1 and paused RNAPII binding at the TESs of TDR genes, which may create a potent barrier to the local assembly of nucleosomes (*42*–*45*) and allow for prompt transcriptional activation over the DoG regions once the PI is lowered by TOP1 loss. Thus, the high levels of pausing and TOP1 that are present over the TDRs could be considered as a means of tuning DoG RNA expression levels by priming the machinery necessary to maintain the potential of TDR genes to become transcribed under different conditions. One possibility by which TOP1 depletion may lower the RNAPII PI at the TES is by leading to the loss of termination factors at TDR genes. It is known that BRD4-dependent phosphorylation of the RNAPII CTD regulates the recruitment of transcription termination factors, including cleavage and polyadenylation specificity factor (CPSF) and cleavage stimulation factor 64 (CSTF64) to Ctrl termination (*46*). Thus, future studies investigating the relationship between BRD4 phosphorylation of RNAPII and its role in both stimulating TOP1 activity and recruiting termination factors will likely provide insights into the direct (causal) effects of TOP1 in regulating termination that we observed in this study. Further supporting this possibility is the recent finding that coinhibition of BRD4 using JQ1 (*47*) and TOP1 using SN38, which is an active metabolite of irinotecan (*48*), disrupts recruitment of CSTF64 at all genes (*26*). However, it is not yet clear from this study whether TOP1 is directly involved in regulating CSTF64 binding and RNAPII-dependent termination. Moreover, additional mechanistic investigations are needed to determine why CSTF64 loss occurred at all genes, but only a subset exhibited readthrough transcription (*26*). Our study reveals that TOP1 directly regulates RNAPII termination and DoG production and that, while a subset of TOP1-dependent DoG RNAs are dependent on its catalytic activity, there is an additional subset that is independent of its catalytic activity. Thus, there are likely several different mechanisms by which TOP1 is regulating termination and DoG production that remain to be further elucidated. Another possibility is that TOP1 loss may favor transcription elongation and DoG production by lowering uncoordinated clashes between replication forks and transcription machinery that can cause replication stress and genomic instability. Consistent with TOP1 depletion lowering the occurrence of transcription-replication collisions (TRCs) is the finding that all replication forks are slowed down by 30 to 40% in TOP1 knockdown cells (*25*). This, together with elongating RNAPII acting as the main obstacle to replication fork progression (*49*), suggests a means in which enhanced DoG production could be favored beyond the ends of genes following TOP1 inhibition. Additional insights are needed to discern the relative importance of topological stress ahead and behind replication forks in the context of TRC-associated replication interference and in DoG production as this remains not well elucidated.

We have uncovered a biological basis for DoG RNA signatures in human cancers through their annotation and characterization in reference to paired NTs for several major cancers that include the colon, breast, and liver. Our study also unveils that these molecular vulnerabilities linked to tumor-promoting genes can be induced under the Ctrl of a regulatory switch governed by an essential regulator of gene Ctrl, TOP1. By defining the previously unrecognized role of TOP1 in regulating termination, we now demonstrate the significance of TOP1 in regulating all stages of the RNAPII transcription cycle and urther support the paradigm that DoG RNAs are increasingly identified as hallmarks of termination defects (*3*, *5*–*7*, *9*, *50*, *51*).

## MATERIALS AND METHODS

### Cell culture and treatments

Human colorectal adenocarcinoma SW480, HCT116, and normal colon FHC cells and human embryonic kidney (HEK) 293T cells were purchased from the American Type Culture Collection. SW480, HCT116, and HEK293T cell lines were cultured in Dulbecco’s modified Eagle’s medium (Gibco) supplemented with 10% fetal bovine serum (Gibco). The FHC cells were grown in Dulbecco’s modified Eagle’s medium:F12 medium (American Type Culture Collection) supplemented with 25 mM Hepes, cholera toxin (10 ng/ml; Sigma-Aldrich, C8052-1MG), insulin (0.005 mg/ml; MP Biomedicals, 0219390025), transferrin (0.005 mg/ml; Sigma-Aldrich, T8158-100MG), hydrocortisone (100 ng/ml; Sigma-Aldrich, H0888-1G), human recombinant epidermal growth factor (20 ng/ml; Thermo Fisher Scientific, PHG0311), and 10% fetal bovine serum (Gibco). All cell lines were grown in a 37°C incubator supplied with 5% CO_2_. All cell lines tested negative for mycoplasma contamination by PCR. SW480 cells were treated with DMSO or 1.15 μM TOP1 poison, CPT-11 (Santa Cruz Biotechnology, CAS 136572-09-3) for 3 hours in a 37°C incubator supplied with 5% CO_2_ before harvesting for RNA expression, cell survival assays, and immunoblot analyses.

### Cell survival assays

To determine the IC_25_ and IC_50_ concentrations for CPT treatment, SW480 cells were treated with 0, 0.1, 1, 2.5, and 5 μM concentrations of CPT diluted in DMSO and incubated at 37°C. After 24 hours, 0.5% crystal violet solution was added to each well and incubated for 20 min at room temperature. The crystal violet solution was washed from the plate with phosphate-buffered saline (PBS), air-dried for 2 hours at room temperature, before methanol was added to each well, and incubated for 20 min at room temperature. Absorbance at 570 nm was measured by TECAN infinite M1000 PRO microplate reader. IC_50_ and IC_25_ were calculated using GraphPad Prism v. 10.

### Lentivirus production and generation of TOP1 knockdown cells

pLKO.1 TRC Ctrl and target shRNA plasmids for TOP1 knockdown were generated with annealed oligos. The shRNA oligos used in this study are listed in table S8. For lentivirus production and transduction, 50 to 60% confluent HEK293T cells were transfected with TRC Ctrl, target shRNA, and packaging plasmids psPAX2 and pMD2.G using Lipofectamine 3000 (Invitrogen). Virus-containing medium was collected 48 and 72 hours after transfection, filtered with a 0.45-μm pore size filter, and used for viral infection. SW480 and HCT116 cells were transduced with viral supernatants containing polybrene (8 μg/ml; Sigma-Aldrich). Following 8 hours, virus-containing medium was removed and replaced with fresh medium. After 48 hours, the cells were selected using puromycin at a final concentration of 1.5 μg/ml before harvesting the cells for qRT-PCR and immunoblot analysis.

### RNA interference experiments using siRNA

SW480 cells were transfected with 100 nM nontargeting siRNA Ctrl or TOP1 siRNA duplexes listed in table S8 (Dharmacon) using Lipofectamine 3000 (Invitrogen) according to the manufacturer’s directions (Life Technologies). Cells were harvested 48 hours after transfection for immunoblot or RNA expression analyses.

### Antibodies

Antibodies used for ChIP assays were obtained commercially as follows: anti-TOP1 (2 μg; Abcam, ab219735), anti-RNAPII (N-20) (2 μg; Santa Cruz Biotechnology, Sc-899), anti–RNA polymerase II subunit B (Rpb1) N-terminal domain (NTD) (D8L4Y) (5 μl; Cell Signaling Technology, #14958), spike-in antibody (2 μg; Active Motif, 61686), and anti–immunoglobulin G (IgG) (2 μg; Cell Signaling Technology, 2729S). Antibodies used for immunoblotting are as follows: anti–β-actin (Santa Cruz Biotechnology, sc47778; 1:1000 dilution), anti-TOP1 (Santa Cruz Biotechnology, sc-32736; 1:1000 dilution), anti-Rpb1 NTD (D8L4Y) (Cell Signaling Technology; #14958; 1:1000 dilution), anti-GAPDH (0411) (Santa Cruz Biotechnology, sc-47724; 1:1000 dilution), and anti-hnRNPA2/B1 (EF-67) (Santa Cruz Biotechnology, sc-53531; 1:1000 dilution). Secondary antibodies used in the study include anti-mouse IgG horseradish peroxidase conjugate (Promega, W402B; 1:3000 dilution) and anti-rabbit IgG horseradish peroxidase Conjugate (Promega, W401B; 1:5000 dilution).

### Tumor tissue analysis

Paired nonneoplastic and tumor tissue was obtained from three colorectal carcinomas through the Department of Pathology of Northwestern University, Feinberg School of Medicine following informed consent from patients. All clinical samples used in this study were reviewed and approved by the Institutional Review Board at the Northwestern University Feinberg School of Medicine. In addition, paired samples of normal and tumor tissue from two patients with breast cancer (R8235086-PP-10) and liver cancer (R8235149-PP-10) were obtained from BioChain Institute Inc. Briefly, for RNA extraction, 1 ml of TRIzol reagent (Invitrogen) was added per 50 mg of tissue into a homogenizing tube (Precellys). For protein extraction, 1 ml of radioimmunoprecipitation assay lysis buffer [10 mM tris-HCl (pH 8.0), 1 mM EDTA, 0.5 mM EGTA, 1% Triton X-100, 0.1% NaDoc, 0.1% SDS, and 140 mM NaCl] was added per 50 mg of tissue into a homogenizing tube (Precellys). Tissues were homogenized with the Bertin Technologies power homogenizer for 10 cycles, 20 s per cycle at speed setting #2. Following homogenization, samples were centrifuged at 12,000*g* for 10 min at 4°C, and the cleared lysate was used for RNA purification or immunoblotting as described in detail below.

#### Cellular fractionation

Cells were washed with ice-cold PBS before lysis in the following cell lysis buffer [10 mM tris (pH 7.4), 150 mM NaCl, 0.15% NP-40, 1 mM phenylmethylsulfonyl fluoride (PMSF), and protease inhibitor cocktail] and incubated on ice for 5 min, before centrifugation at 3500*g* for 10 min. The supernatant containing the cytoplasmic fraction was cleared by centrifugation at 14,000*g* for an additional 1 min in a fresh tube. The isolated nucleus pellet was rinsed with 1 ml of ice-cold PBS with 500 mM EDTA. Each fraction was divided for protein lysate preparation and RNA extraction.

### Western blotting

Protein samples were denatured at 95°C for 5 min, separated by SDS–polyacrylamide gel electrophoresis, and transferred to polyvinylidene difluoride membranes using the iBlot2 gel transfer device (Invitrogen). The membranes were blocked in 3% milk and probed with the indicated antibodies. Reactive bands were detected by Pierce ECL Plus (Thermo Fisher Scientific) or SuperSignal West Femto (Thermo Fisher Scientific) and visualized using the Odyssey Fc Imaging System (LI-COR Biosciences) or the ChemiDoc Imaging system (Bio-Rad Laboratories).

### RNA purification and qRT-PCR

Total RNA was extracted with TRIzol reagent (Invitrogen) from paired patient tissues (breast, colon, and liver), SW480, HCT116, and FHC cells; SW480 and HCT116 cells stably expressing Ctrl or TOP1 shRNAs; or SW480 cells transiently expressing Ctrl or TOP1 siRNAs following the manufacturer’s instructions. Total RNA was used for cDNA synthesis using the ProtoScript II First Strand cDNA Synthesis Kit [New England Biolabs (NEB)] with random hexamers. PCR reactions were performed using SYBR Green PCR Master Mix (Applied Biosystems) on an Applied Biosystems QuantStudio3 real-time PCR system and iTaq Universal SYBR Green Supermix (Bio-Rad Laboratories) on CFX Opus 384 Real-Time PCR System (Bio-Rad Laboratories). The specificity of amplification was confirmed by melting curve analysis. The relative levels of DoG and mRNA expression were calculated using the ΔΔ*C*_t_ method normalized to 18*S* rRNA (tumor samples) or GAPDH (cell lines). The expression levels in TOP1 knockdown are relative to the Ctrl knockdown. Primers used for qRT-PCR are listed in table S8.

### RNA sequencing

Total RNA from three paired patient tissues (COAD and NTs), HCT116, SW480, and FHC cells, and from SW480 cells stably expressing Ctrl or TOP1 shRNAs or treated with CPT versus DMSO was extracted using TRIzol reagent (Invitrogen) and Direct-zol RNA Microprep kit (Zymo Research) according to the manufacturer’s instructions. Total RNA-seq (ribo-depleted) libraries for all samples were generated and sequenced by Admera Health. Briefly, isolated RNA sample quality was assessed by High Sensitivity RNA TapeStation (Agilent Technologies, CA, USA) and quantified by Qubit 2.0 RNA HS assay (Thermo Fisher Scientific, MA, USA). Libraries were constructed with KAPA RNA HyperPrep with RiboErase (Roche, IN, USA) and performed on the basis of the manufacturer’s recommendations. Final library quantity was measured by KAPA SYBR FAST qPCR and library quality evaluated by TapeStation D1000 ScreenTape (Agilent Technologies, CA, USA). The final library size was about 430 bp with an insert size of about 200 bp. Illumina 8–nucleotide (nt) dual indices were used. Equimolar pooling of libraries was performed based on quality Ctrl (QC) values and sequenced on an Illumina NovaSeq S4 (Illumina, CA, USA) with a read length configuration of 150 paired-end (PE) for 50 M PE reads per sample (25 M in each direction).

### TOP1 sequencing

The TOP1-seq methodology was adapted from a previously established protocol (*22*). Briefly, 24 × 10^6^ SW480 cells were treated with 10 μM CPT for 4 min. Cells were immediately washed with ice-cold 1× PBS, scraped, resuspended in lysis buffer [20 mM tris-HCl (pH 7.5), 300 mM NaCl, 2 mM EDTA, 0.5% NP-40, 1% Triton X-100, 1 mM PMSF, and PICs], and incubated on ice for 15 min. Cell resuspensions were dounced 10 times using an ice-cold glass homogenizer. Nuclei were collected by centrifugation and resuspended in shearing buffer [0.1% SDS, 0.5% *N*-lauroylsarcosine, 1% Triton X-100, 10 mM tris-HCl (pH 8.1), 100 mM NaCl, 1 mM EDTA, 1 mM PMSF, and PICs]. Chromatin was fragmented using a Bioruptor Pico sonicator (Diagenode) to an average size of 200 to 600 bp, and cleared chromatin was used for immunoprecipitation overnight at 4°C with protein A Dynabeads that were precoupled in 0.5% bovine serum albumin with IgG or TOP1 antibodies. The immunocomplex-bound beads were washed eight times in wash buffer [50 mM Hepes-KOH (pH 7.6), 500 mM LiCl, 1 mM EDTA, 1% NP-40, 0.7% sodium deoxycholate, 1 mM PMSF, and PICs], twice in 1× TE, and eluted in elution buffer [50 mM tris-HCl (pH 8.0), 10 mM EDTA, and 1% SDS]. Immunoprecipitated DNA samples were purified using UPrep Spin Columns (Genesee Scientific) and quantified with a Qubit 4.0 fluorometer (Invitrogen). We used 10 ng of DNA from two biological replicates to generate sequencing libraries using KAPA Hyper Prep Kit (KAPA Biosystems) according to the manufacturer’s instructions. Briefly, TOP1-seq was subjected to end repair and adaptor ligation, followed by indexed PCR. Libraries were size-selected for average size of 300 bp. Sequencing was performed on an Illumina NovaSeq 6000 (Illumina, CA, USA) with a read length configuration of 75 single-end (SE) per sample.

### Precision nuclear run-on sequencing

PRO-seq was performed following established protocol (*52*). Nuclei from SW480 cells were isolated and snap-frozen in storage buffer [10 mM tris-HCl (pH 8.0), 25% glycerol, 5 mM MgCl_2_, 0.1 mM EDTA, and 5 mM dithiothreitol (DTT)]. Next, 10 × 10^6^ nuclei and 100 μl of storage buffer were run on by the addition of 100 μl of prewarmed 2× reaction buffer [10 mM tris-HCl (pH 8.0), 300 mM KCl, 5 mM MgCl_2_, 1% sarkosyl, 1 mM DTT, RNaseOUT (0.8 U/μl; Thermo Fisher Scientific), 250 μM adenosine 5′-triphosphate, 250 μM guanosine 5′-triphosphate, 50 μM biotin-11–cytidine 5′-triphosphate (PerkinElmer), and 50 μM biotin-11–uridine 5′-triphosphate (PerkinElmer)] for 3 min at 37°C. Reactions were stopped by the addition of 500 μl of TRIzol LS (Invitrogen) and purified following the manufacturer’s instructions. RNAs were heat-denatured at 65°C for 40 s, placed on ice, and fragmented with 0.2 N of NaOH on ice for 10 min. After pH adjustment with 1 M tris-HCl (pH 6.8), buffer exchange using P-30 columns (Bio-Rad Laboratories) was performed. Biotinylated RNAs were enriched with 30 μl of prewashed Dynabeads MyOne Streptavidin C1 (Life Technologies) in binding buffer [10 mM tris-HCl (pH 7.4), 300 mM NaCl, 0.1% Triton X-100, and RNaseOUT (0.8 U/μl)] and by gentle rotation at room temperature for 30 min. Beads were subsequently washed twice with ice-cold high-salt wash buffer [50 mM tris-HCl (pH 7.4), 2 M NaCl, and 0.5% Triton X-100], twice with binding buffer, and once with low-salt wash buffer [5 mM tris-HCl (pH 7.4) and 0.1% Triton X-100] and eluted with TRIzol LS (Invitrogen). The 3′ RNA adaptor (VRA3: 5′-GAUCGUCGGACUGUAGAACUCUGAAC-/inverted dT/-3′) was ligated to the eluted RNAs, and a second biotin RNA enrichment was performed as described above. Next, the 5′ end of RNAs was enzymatically modified with RNA 5′ pyrophosphohydrolase (NEB), and hydroxyl repair was performed using T4 polynucleotide kinase (NEB). Purified RNAs were subsequently ligated with the 5′ RNA adaptor (RA5: 5′-CCUUGGCACCCGAGAAUUCCA-3′). A third biotin RNA enrichment was performed before reverse transcription of the eluted RNAs. Libraries from two biological replicates were amplified for 13 cycles, size-selected for 140 to 350 bp, and single-end sequenced on Illumina HiSeq 4000 (75 bp).

### Chromatin immunoprecipitation

TOP1 and RNAPII ChIP assays were performed using 24 × 10^6^ to 48 × 10^6^ SW480 cells, and RNAPII ChIP assays were performed using the same number of SW480 cells stably expressing Ctrl or TOP1 shRNAs. The cells were cross-linked with 2 mM disuccinimidyl glutarate (ProteoChem) for 30 min, followed by cross-linking with 1% formaldehyde for an additional 10 min at room temperature and quenched with 125 mM glycine (Thermo Fisher Scientific) for 5 min. Cell pellets were resuspended in lysis buffer [20 mM tris-HCl (pH 7.5), 300 mM NaCl, 2 mM EDTA, 0.5% NP-40, 1% Triton X-100, 1 mM PMSF, and PICs] and incubated on ice for 30 min. Cell resuspensions were next dounced 10 times in a prechilled glass homogenizer. Nuclei were collected and resuspended in shearing buffer [0.1% SDS, 0.5% *N*-lauroylsarcosine, 1% Triton X-100, 10 mM tris-HCl (pH 8.1), 100 mM NaCl, 1 mM EDTA, 1 mM PMSF, and PICs]. Chromatin was fragmented using a Bioruptor Pico (Diagenode) or an E220 focused ultrasonicator (Covaris) to an average size of 200 to 600 bp, and the cleared chromatin was used for immunoprecipitation overnight at 4°C with Protein A Dynabeads that were precoupled in 0.5% bovine serum albumin with IgG, TOP1, RNAPII, and spike-in antibodies. Spike-in chromatin (Active Motif, 53083) was added to normalize out technical variation. The immunocomplex-bound beads were washed eight times in wash buffer [50 mM Hepes-KOH (pH 7.6), 500 mM LiCl, 1 mM EDTA, 1% NP-40, 0.7% sodium deoxycholate, 1 mM PMSF, and PICs] and twice in 1× TE and eluted in elution buffer [50 mM tris-HCl pH 8.0, 10 mM EDTA, and 1% SDS]. Cross-links were reversed at 65°C overnight, and the DNA samples were purified using UPrep Spin Columns (Genesee Scientific).

ChIP-seq libraries for all samples were generated and sequenced by Admera Health. Briefly, immunoprecipitated DNA was quantified with Qubit 2.0 DNA HS Assay (Thermo Fisher Scientific, MA, USA) and quality-assessed by TapeStation genomic DNA Assay (Agilent Technologies, CA, USA). Library preparation was performed using KAPA Hyper Prep (Roche, Basel, Switzerland) following the manufacturer’s recommendations. All samples were subjected to end repair and adaptor ligation, followed by indexed PCR using Illumina 8-nt dual-indices. Library quality and quantity were assessed with Qubit 2.0 DNA HS Assay and TapeStation High Sensitivity D1000 Assay (Agilent Technologies, CA, USA). Final libraries were quantified using QuantStudio 5 System (Applied Biosystems, CA, USA) before equimolar pooling based on qPCR QC values. Sequencing was performed on an Illumina NovaSeq (Illumina, CA, USA) with a read length configuration of 150 PE for 40 M PE reads 20 M in each direction) per sample.

### Coimmunoprecipitation

Coimmunoprecipitation was performed following established protocol (*53*). Briefly, 12 × 10^6^ cells were lysed in lysis buffer [10 mM tris-HCl (pH 7.4), 10 mM KCl, 1.5 mM MgCl_2_, 12% sucrose, 10% glycerol, 0.2% Triton X-100, 0.5 mM DTT, 1 mM PMSF, PICs, and phosphatase inhibitors] and then fractionated in sucrose cushion [10 mM tris-HCl (pH 7.4), 10 mM KCl, 1.5 mM MgCl_2_, 30% sucrose, and 0.5 mM DTT]. Nuclei were stored in freeze buffer [10 mM tris-HCl (pH 7.4), 10 mM KCl, 1.5 mM MgCl_2_, 40% glycerol, and 0.5 mM DTT] at −80°C. Chromatin DNA was digested in chromatin digestion buffer [20 mM tris-HCl (pH 7.4), 150 mM NaCl, 1.5 mM MgCl_2_, 10% glycerol, 0.05% NP-40, 0.5 mM DTT, 1 mM PMSF, PICs, phosphatase inhibitors, and benzonase (250 U/ml)]. After centrifugation, the supernatant was collected as fraction 1, while proteins were further extracted from the pellet in chromatin-2 buffer [20 mM tris-HCl (pH 7.4), 500 mM NaCl, 1.5 mM MgCl_2_, 10% glycerol, 0.05% NP-40, 0.5 mM DTT, 1 mM PMSF, PICs, phosphatase inhibitors, and 3 mM EDTA] as fraction 2. NaCl concentration in fraction 2 was adjusted to 150 mM and then combined with fraction 1. TOP1 immunoprecipitation was performed using anti-TOP1 (2 μg; Abcam, ab219735), conjugated to Protein A Dynabeads for 24 hours. The immunocomplex-bound beads were washed four times in wash buffer [20 mM tris-HCl (pH 7.4), 225 mM NaCl, 1.5 mM MgCl_2_, 10% glycerol, 1.5 mM EDTA, 0.05% NP-40, and 0.5 mM DTT], and immunoprecipitated proteins were eluted in Laemmli SDS sample buffer before immunoblotting analysis for TOP1 and RNAPII.

### Total RNA-seq analysis

The QC of raw data was evaluated with FastQC (v0.11.5) (*54*) and MultiQC (*55*). The reads were trimmed for adapter contaminations and quality using Trim_Galore (v0.6.10 with cutadapt v4.2) (*56*) with the following parameters: “--clip_R1 15 --clip_R2 15 --three_prime_clip_R1 5 --three_prime_clip_R2 5.” Paired-end raw reads were mapped to the hg38 human genome with GENECODE v43 (primary_assembly) using STAR aligner v2.7.9a (*57*) with the following parameters: “--genomeLoad NoSharedMemory --outFilterMultimapNmax 20 --alignSJoverhangMin 8 --alignSJDBoverhangMin 1 --outFilterMismatchNmax 999 --outFilterMismatchNoverReadLmax 0.04 --alignIntronMin 20 --alignIntronMax 1000000 --alignMatesGapMax 1000000 --outSAMunmapped Within --outFilterType BySJout --outSAMattributes NH HI AS NM MD --outSAMtype BAM SortedByCoordinate --quantMode TranscriptomeSAM GeneCounts --sjdbScore 1 --limitBAMsortRAM 60000000000 --outWigType wiggle --outWigNorm None.” Only high-quality reads (both ends were uniquely mapped, passed quality check, and were with mapping quality score ≥ 255) were kept for the analysis. The gene expression was determined with featureCounts (subread v1.6.1) (*58*) using GENECODE v43 (primary_assembly) gene annotation with the parameters: “-s 2 -B -p -t exon -g gene_id” (*58*) The gene counts were used to determine the differential gene expression with DESeq2 v1.36 (*59*) and *q* < 0.05 and a log_2_ FC of 0.58. Volcano plots were generated using ggplot2 (v3.4.3) (*60*). The gene set enrichment analysis was carried out using enrichr [Pathway- > Molecular Signatures Database (MSigDB) Hallmark 2020] (*61*). Terms with a *P* < 0.05 were deemed statistically significant. The box plot, bar plot, dot plot, Venn diagram, and statistical analysis were performed in the ggplot2 package of R (v4.2.0) (*60*).

### DoG RNA identification

BAM files from our total RNA-seq analysis aligned to the hg38 human genome were sorted and indexed with SAMtools v1.14 (*62*). DoG RNAs were identified using DoGFinder (*33*) with the following parameters, Get_DoGs “-minDoGLen 5000 -minDoGCov 0.6 -w 200 -mode F” using the protein-coding genes GENCODE v43 annotation. “-S” was added for our own RNA-seq data due to the reverse-stranded specific protocol but not in TCGA RNA-seq data (unstranded protocol). Samples within each group were merged to increase the coverage of DoG calling. Downsampling of the following RNA-seq datasets—(i) NT and COAD group, (ii) cell lines including the SW480, HCT116 and FHC, and (iii) the shTOP1 and shCtrl cell lines—was performed to reduce the differences in sequencing depth using the Pre_Process function in DoGFinder. Only DoGs derived from protein-coding genes (GENCODE v43, primary_assembly) were kept for analysis. Further, within 5-kb downstream of DoG host genes’ TES, if there is any other gene annotation [GENCODE v43 or National Center for Biotechnology Information RefSeq annotation from University of California, Santa Cruz (UCSC), same strand as DoG hosting genes for our own stranded specific RNA-seq or no strand requirement for TCGA unstranded RNA-seq], these DoGs will be filtered out for the downstream analysis to avoid the quantification of other genes’ expression into DoG’s expression. The DoG coordinates and strand information provided by DoGFinder were converted to GTF format by bedToGenePred and genePredToGtf (UCSC kentUtils 302) (*63*) and used to quantify the DoG’s expression level. The gene expression of DoGs was quantified with featureCounts (subread v1.6.1) (*58*) on these coordinates with the parameters: “-s 2 -B -p -t exon -g gene_id” (-s 0 for TCGA RNA-seq due to its unstranded protocol) to identify the counts and further generate BigWig files for visualization. TPM normalization method was used, and the log_2_ ratio between samples was calculated (COAD samples over NT samples, SW480 and HCT116 over FHC cells, and shTOP1 over shCtrl cells). Box plots were generated using ggplot2 (*60*). The adjacent genes were identified by the closest function available in bedtools v2.25.0 (*64*).

### TCGA data analysis

To identify DoG RNA signatures in breast, colon, and liver paired NTs and tumors, RNA-seq data from a total of 44 patient tissues (22 paired tumors and NTs) were used from the TCGA database (*32*, *35*). STAR-aligned RNA-seq BAM files of patients with BRCA, COAD, and LIHC from TCGA were downloaded from the Genomic Data Commons with the dbGaP accession phs000178.v10.p8. For this study, TCGA samples that were prepared for RNA-seq by polyadenylation captured RNA libraries, and only paired-end format files were used. Only high-quality reads (both ends were uniquely mapped, passed quality check, and were with mapping quality score ≥ 255) were kept for the analysis. The gene expression was determined with featureCounts (subread v1.6.1) (*58*) using GENECODE v43 (primary_assembly) gene annotation with the parameters: “-s 0 -B -p -t exon -g gene_id.” The gene counts were used to determine the differential gene expression with DESeq2 v1.36 (*59*) and *q* < 0.05 and a log_2_ FC of 0.58. Volcano plots were generated using ggplot2 (v3.4.3) (*60*) and DoGFinder (*33*) using Get_DoGs -minDoGLen 5000 -minDoGCov 0.6 -w 200 -mode F using the GENCODE v43 annotation (primary assembly). TOP1 levels in patients with COAD were determined and ranked by increasing values.

Kaplan-Meier curves were generated by the OS time in days of patients from TCGA downloaded from UCSC Xena survival package (v3.5-7) from R (v4.2.0) (*65*) The median DoG number and extension strength for BRCA, COAD, and LIHC were used as the cutoff value. The significance was determined by a log-rank test in survival package (v3.5-7) from R.

To identify differentially expressed TOP1 levels in COAD versus NTs, we used the RNA-seq data available for 275 COAD and 349 NTs from TCGA and GTEx databases through GEPIA website (http://gepia.cancer-pku.cn/detail.php?gene=top1) (*32*, *35*, *66*). To identify differentially expressed *TOP1* mRNA levels in the breast, colon, and liver, RNA-seq data from TCGA were downloaded from the Genomic Data Commons with the dbGaP accession phs000178.v10.p8. RNA-seq data was analyzed from a total of 44 patient tissues (22 paired tumors and NTs) from BRCA, COAD, and LIHC.

### ChIP-seq and TOP1-seq analysis

The QC of raw data was evaluated with FastQC (v0.11.5) (*54*). The low-quality reads were removed using Trim_Galore (v0.6.10 with cutadapt v4.2) (*56*) with the following parameters: “--clip_R1 3 --clip_R2 3 --three_prime_clip_R1 3 --three_prime_clip_R2 3” for paired-end reads and “--clip_R1 3 --three_prime_clip_R1 3” for single-end reads. Sequencing reads were mapped to the hg38 human genome (GCA_000001405.15_GRCh38_no_alt_analysis_set.fa) using bwa v0.7.17 (mem for paired-end reads and aln and samse for single-end reads) (*67*) and default parameters. SAMtools v1.14 (*62*) was used for filtering the mapping quality and the duplicate reads. Only high-quality reads (uniquely mapped, passed quality check, no PCR duplicates, and with mapping quality score ≥ 30). For paired end reads, we further required properly paired and both ends uniquely mapped) were used for further analysis. Spike-in normalization was performed by mapping the reads to the *Drosophila melanogaster* genome (dm6) using bwa-mem with the same parameters. deepTools v3.5.1 (*68*) was used to generate normalized BigWig files by combining reads from two replicas. The bins per million mapped normalized reads were used, and the log_2_ ratio between the ChIP-seq signal over input was calculated. The BigWig files were visualized in Integrative Genomics Viewer (IGV) (*69*).

### PRO-seq analysis

The QC of raw data was evaluated with FastQC (v.0.11.5) (*54*). The low-quality reads were removed using Trim_Galore (v0.6.10 with cutadapt v4.2) (*56*) with the following parameters: “--clip_R1 10 --clip_R2 3 --three_prime_clip_R1 2 --three_prime_clip_R2 2.” Sequencing reads were mapped to the hg38 human genome (GCA_000001405.15_GRCh38_no_alt_analysis_set.fa) using bwa-mem (v0.7.17) with default parameters. SAMtools v1.14 (*62*) was used for filtering the mapping quality and the duplicate reads. Only high-quality reads (both ends uniquely mapped, passed quality check, no PCR duplicates, properly paired, and with mapping quality score ≥ 30) were used for further analysis. deepTools (v3.5.1) (*68*) was used to generate normalized BigWig files.

### TSS and TES PI

To calculate the TSS PI, we used the previously described method (*70*). bigWigAverageOverBed from UCSC tools (*68*) was used to summarize the coverage of RNAPII counts (RNAPII ChIP-seq) or PRO-seq counts at promoters of TDR genes (−50 to +300 bp around TSS) and the gene body (+300 downstream to the annotated gene end). The PI was calculated by the following formula (Fig. 5D):

$${\text{PI}}_{\text{TSS}}=(\text{read counts at TSS}/\text{length1})/(\text{read counts at gene body}/\text{length2})$$

where “length1” is the length of the promoter region (350 bp) and “length2” is the length of the gene body region (+300 downstream of the TSS to the annotated gene end). A PI of 2 was used to define the paused TDR genes.

To define the TES PI we used a similar method as was used for calculating the TSS PI. bigWigAverageOverBed from UCSC tools (*68*) was used to analyze the coverage of RNAPII counts (RNAPII ChIP-seq or PRO-seq) at +500 to +1500 bp around TES and +10,000 downstream to the annotated gene end for non-DoG–producing genes. For TDRs and DoGs in SW480 cells, we used the region spanning from +1500 downstream to the annotated gene end to the end of the DoG defined by DoGFinder (*33*). The region at +500 to +1500 bp around the TES was defined according to RNAPII enrichment at TES regions (Fig. 5C). Figure 5D shows an illustration of the TSS and TES pausing indices calculated from PRO-seq.

### Statistical analysis

All statistical tests were performed using GraphPad Prism version 8 or 9 or statistical functions in R or Python using the tests described for each experiment. Log_2_ FC > 0.58 or log_2_ FC < −0.58, *q* < 0.05, and *P* < 0.05. Information about the statistical tests is provided in the figure legends for the respective figures and relevant subsections in Materials and Methods.
