# Supplementary material for: Downstream-of-gene (DoG) transcripts contribute to an imbalance in the cancer cell transcriptome
Source: Sci Adv. 2024 Jul 3;10(27):eadh9613. doi: 10.1126/sciadv.adh9613 (PMC11221514; doi:10.1126/sciadv.adh9613)
Supplement: Supplementary file 1 — Figs. S1 to S5 Legends for tables S1 to S8 [file sciadv.adh9613_sm.pdf]

Science Advances
AAAS

Supplementary Materials for

Downstream-of-gene (DoG) transcripts contribute to an imbalance in the cancer cell transcriptome

Kouki Abe *et al.*

Corresponding author: Shannon M. Lauberth, shannon.lauberth@northwestern.edu;
Yaping Liu, yaping@northwestern.edu

Sci. Adv. **10**, eadh9613 (2024)
DOI: 10.1126/sciadv.adh9613

The PDF file includes:

Figs. S1 to S5
Legends for tables S1 to S8

Other Supplementary Material for this manuscript includes the following:

Tables S1 to S8

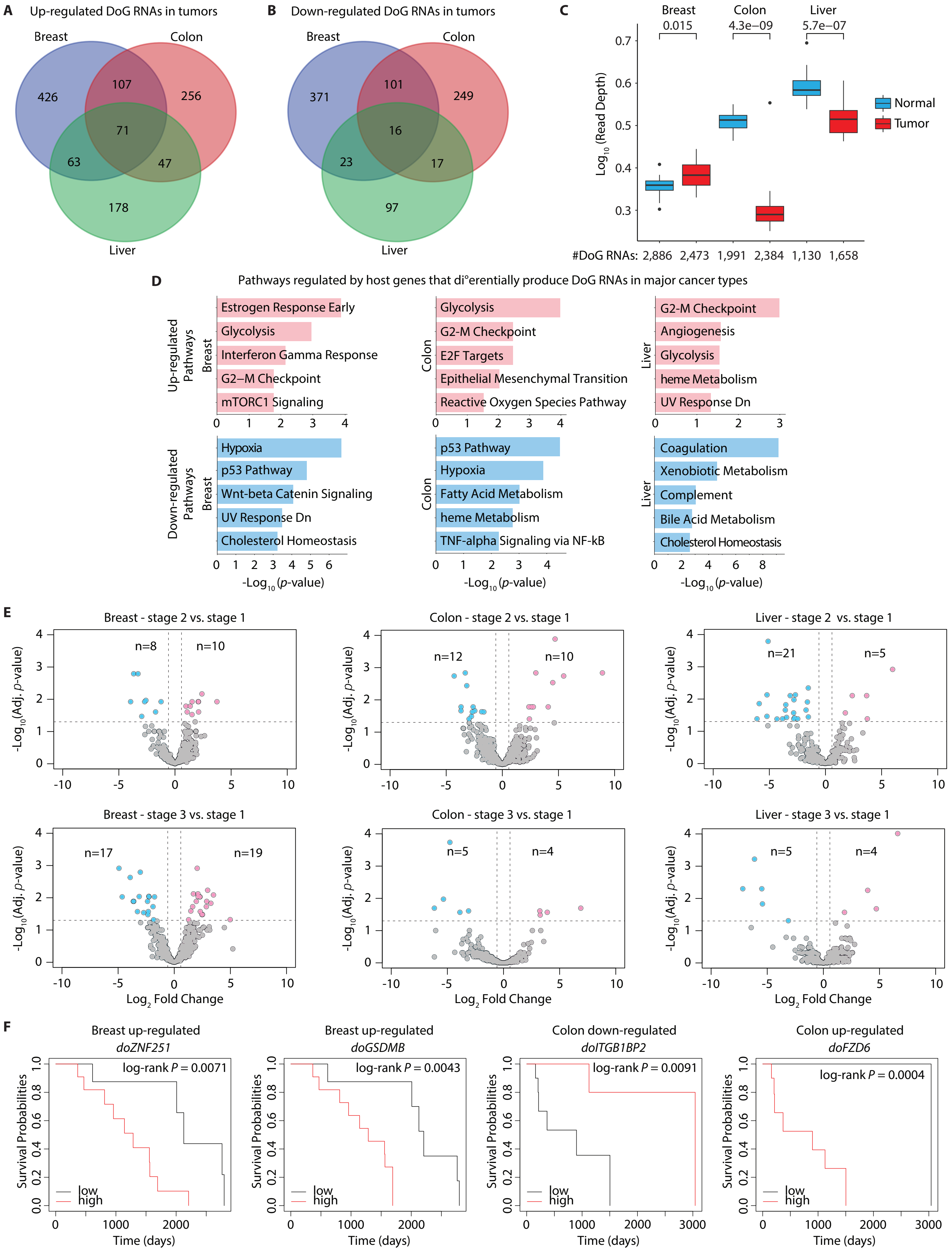
A
Up-regulated DoG RNAs in tumors
Breast
Colon
Liver
426
107
256
71
63
47
178
B
Down-regulated DoG RNAs in tumors
Breast
Colon
Liver
371
101
249
16
23
17
97
C
Breast
0.015
Colon
4.3e−09
Liver
5.7e−07
Log10 (Read Depth)
Normal
Tumor
#DoG RNAs: 2,886 2,473 1,991 2,384 1,130 1,658
D
Pathways regulated by host genes that di°erentially produce DoG RNAs in major cancer types
Up-regulated Pathways
Breast
Estrogen Response Early
Glycolysis
Interferon Gamma Response
G2−M Checkpoint
mTORC1 Signaling
Colon
Glycolysis
G2-M Checkpoint
E2F Targets
Epithelial Mesenchymal Transition
Reactive Oxygen Species Pathway
Liver
G2-M Checkpoint
Angiogenesis
Glycolysis
heme Metabolism
UV Response Dn
Down-regulated Pathways
Breast
Hypoxia
p53 Pathway
Wnt-beta Catenin Signaling
UV Response Dn
Cholesterol Homeostasis
Colon
p53 Pathway
Hypoxia
Fatty Acid Metabolism
heme Metabolism
TNF-alpha Signaling via NF-kB
Liver
Coagulation
Xenobiotic Metabolism
Complement
Bile Acid Metabolism
Cholesterol Homeostasis
-Log10 (p-value)
E
Breast - stage 2 vs. stage 1
n=8
n=10
Colon - stage 2 vs. stage 1
n=12
n=10
Liver - stage 2 vs. stage 1
n=21
n=5
Breast - stage 3 vs. stage 1
n=17
n=19
Colon - stage 3 vs. stage 1
n=5
n=4
Liver - stage 3 vs. stage 1
n=5
n=4
-Log10(Adj. p-value)
Log2 Fold Change
F
Breast up-regulated
doZNF251
log-rank P = 0.0071
Breast up-regulated
doGSDMB
log-rank P = 0.0043
Colon down-regulated
doITGB1BP2
log-rank P = 0.0091
Colon up-regulated
doFZD6
log-rank P = 0.0004
Survival Probabilities
low
high
Time (days)

Fig. S1. Common and specific DoG RNAs in breast, colon, and liver.

A, Venn diagram showing the overlap of upregulated DoG RNAs in breast, colon, and liver tumors. **B**, Venn diagram showing the overlap of downregulated DoG RNAs in breast, colon, and liver tumors. **C**, Ratios of mRNAs with and without a DoG RNA in NTs and BRCA, COAD, and LIHC tumors. The read depth is shown in Log10 scale. **D**, Top five (p-value<0.05) MSigDB pathways for (top) upregulated and (bottom) downregulated DoG RNAs in breast, colon, and liver tumors compared with paired normal tissues. **E**, Volcano plots of stage-specific differentially expressed DoG RNAs (Log_2 FC>0.58 or Log_2 FC<-0.58, q-value< 0.05) with (top) stage 2 versus 1 and (bottom) stage 3 versus 1 of breast (left,) colon (middle), and liver tumors (right). **F**, Kaplan-Meier survival plots with differentially expressed late-stage DoG RNAs associated with poor patient survival in breast. (left) Upregulated stage 3-specific DoG RNAs in BRCA tumors are shown and include (*doZNF251*, *doGSDMB*) and (right) stage 2-specific downregulated DoG RNA (*doITGB1BP2*) and stage 2-specific upregulated DoG RNA (*doFZD6*) are shown. Statistical significance was determined using a Log-Rank test.

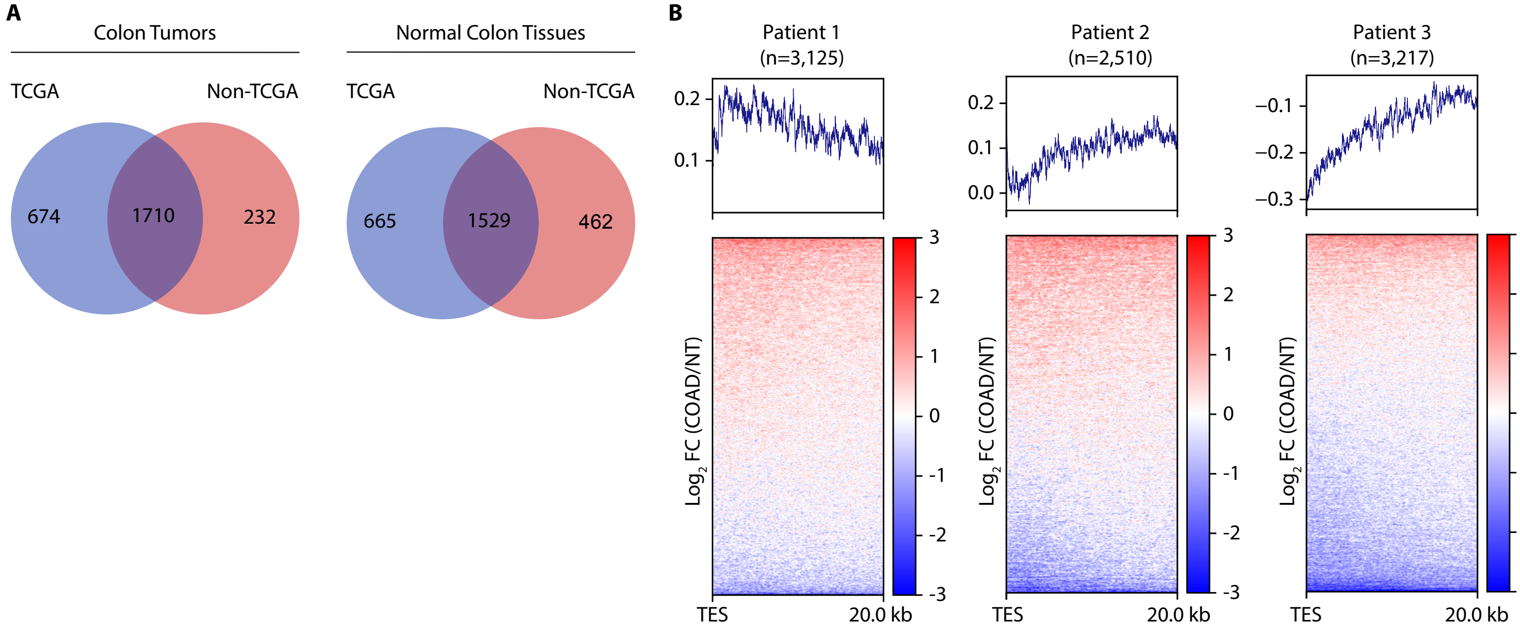

Fig. S2. DoG RNA production in colorectal carcinoma tumors.

A, Venn diagram showing the overlap of DoG RNAs in TCGA versus non-TCGA (left) COADs and (right) normal colon tissues. **B**, Heatmaps of the Log_2-transformed fold change in RNA-seq signal in COADs relative to paired NTs from three independent patients. RNA-seq signal is shown as Reads Per Kilobase Million (RPKM) spanning from the TES to 20.0 kb downstream of all annotated genes.

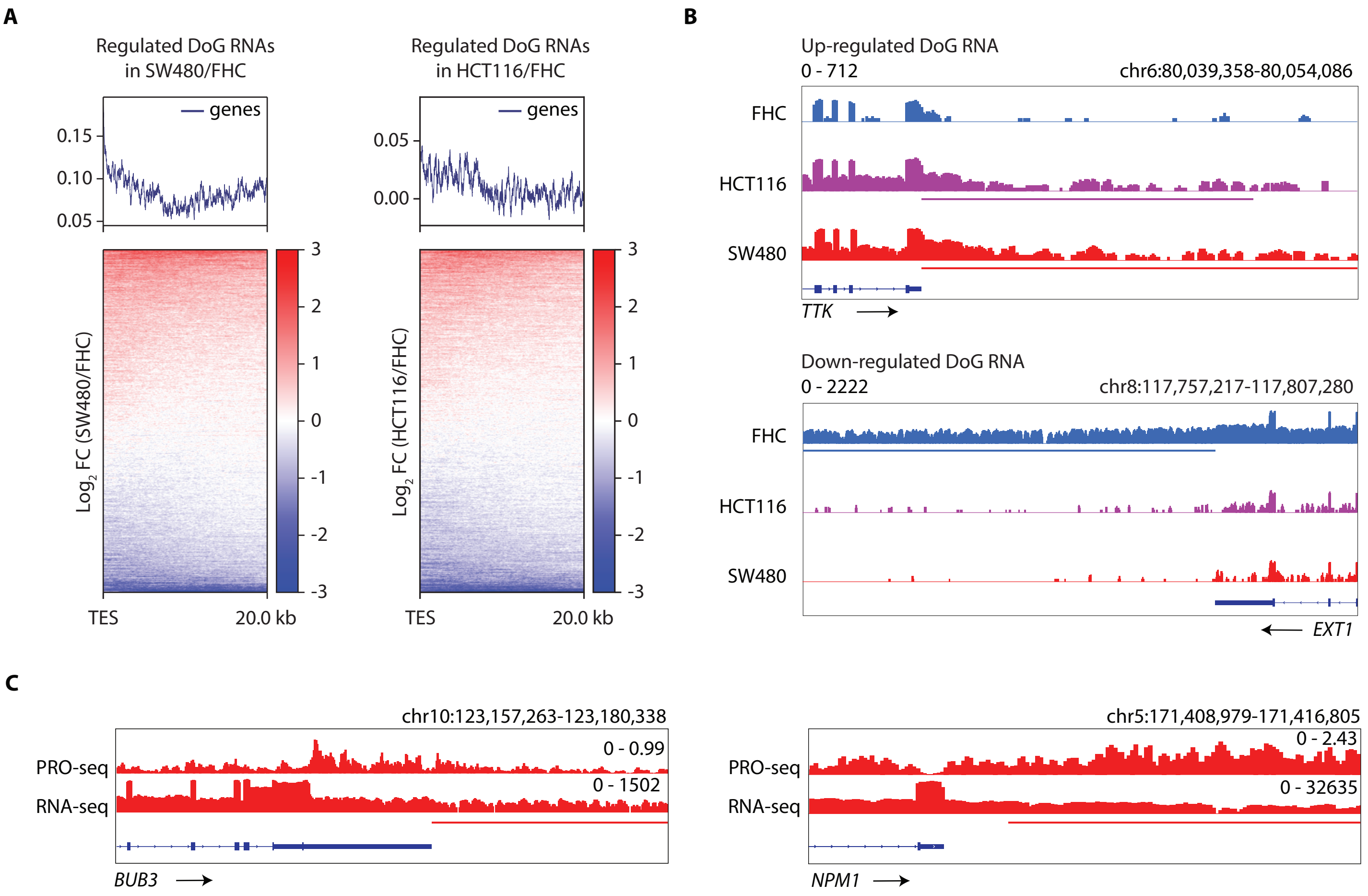

Fig. S3. DoG RNAs in colorectal carcinoma cell lines.

A, Heatmaps of the Log_2-transformed fold change in RNA-seq signal (SW480/FHC and HCT116/FHC) in Reads Per Kilobase Million (RPKM) spanning from the TES to 20.0 kb downstream of all annotated genes. **B**, IGV tracks of total RNA-seq signal in Log RPKM of *TTK* and *EXT1* loci in FHC, HCT116, and SW480 cells. The horizontal bars define the DoG region determined by DoGFinder (*32*). **C**, IGV tracks of PRO-seq and RNA-seq signal in Log RPKM in SW480 cells of *BUB3* and *NPM1* loci spanning the TES to the predicted end of the DoG as defined by DoGFinder (*32*). The horizontal bar defines the DoG regions identified by DoGFinder (*32*).

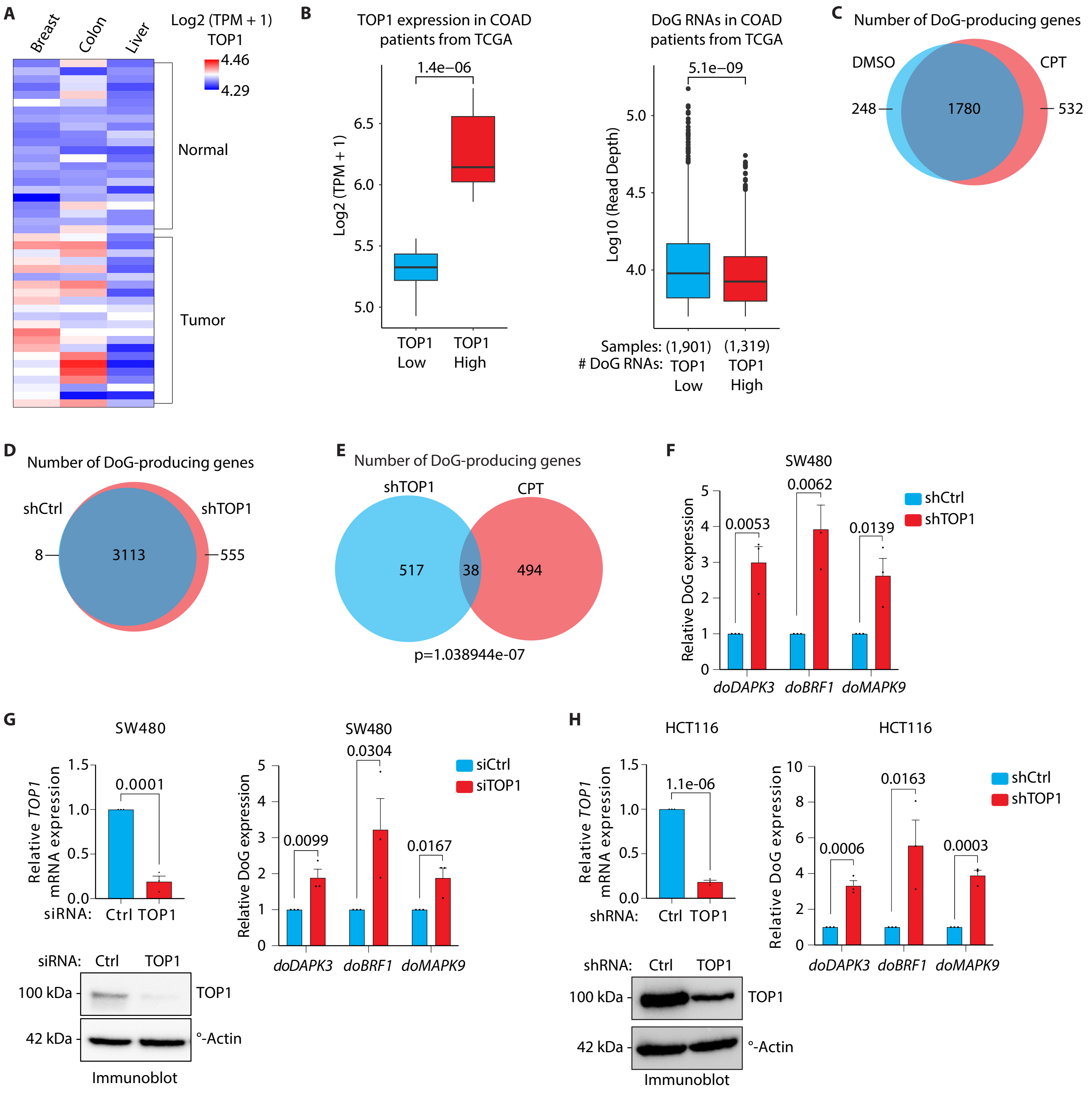

Fig. S4. Inhibition and Depletion of TOP1 leads to potent DoG RNA production.

A, Heatmap showing TOP1 expression levels in breast, colon, and liver tumors (n=22) and their matched normal tissues (n=22) from TCGA. *TOP1* mRNA expression levels shown in Log2 (TPM+1) in COADs versus NTs (*p*=2.82e-08) and BRCA versus NTs (*p*=0.001468). Statistical significance was determined by two-sided Mann-Whitney U test. **B**. Box plot for *TOP1* mRNA (left) and DoG RNA (right) expression levels shown in Log2 (TPM +1) in COADs with low *TOP1*

(blue, n=11) or high *TOP1* mRNAs levels (red, n=11) from TCGA(*31, 34, 65*) Boxplots enclose values between first and third quartiles, midlines show medians, and whiskers extend to data points within 1.5 the interquartile range from the box, outliers are shown. Statistical significance was determined by one-way Wilcoxon rank-sum test (alternative=”less”) (**p*-value < 0.05). **C,** Venn diagram showing overlap of DoG-producing genes in SW480 cells treated with DMSO or CPT. **D,** Venn diagram showing the overlap of DoG-producing genes in SW480 cells expressing Ctrl versus TOP1 shRNA. **E,** Venn diagram showing the overlap of DoG-producing genes in SW480 cells following treatment with CPT versus expressing TOP1 shRNA. **F,** qRT-PCR analysis of the expression levels of the following DoG RNAs, *doDAPK3, doBRF1, and doMAPK9* in SW480 cells expressing Ctrl and TOP1 shRNA. Expression levels are relative to Ctrl shRNA. Data represents the mean and s.e.m. of three independent replicates. *p*-values are shown in figure panel. **G,** (left) qRT-PCR and immunoblot analysis of *TOP1* mRNA and TOP1 protein levels, respectively in SW480 cells transfected with Ctrl or TOP1 siRNA. qRT-PCR data represents the mean and s.e.m. of three independent replicates. *p*-value = 0.0001. The western blot image is representative of three independent images and β-Actin was used as loading control. (right) qRT-PCR analysis of DoG RNAs, *doDAPK3, doBRF1,* and *doMAPK9* in SW480 cells transfected with Ctrl and TOP1 siRNA. Expression levels are relative to Ctrl siRNA. Data are shown as mean ± SE, n = 3. *p*-values are shown on the bar graph. H**,** (left) qRT-PCR and immunoblot analysis of TOP1 in HCT116 cells transfected with Ctrl and TOP1 shRNA (n=3). β-Actin was used as loading control. Data are shown as mean ± SE, n=3. *p*-values are shown on the bar graph. (right) qRT-PCR analysis of the following DoG RNAs, *doDAPK3*, *doBRF1*, and *doMAPK9* in HCT116 cells infected with Ctrl or TOP1 s**h**RNA. Expression levels are relative to Ctrl shRNA. Data are shown as mean ± SE, n = 3. *p*-values are shown on the bar graph.

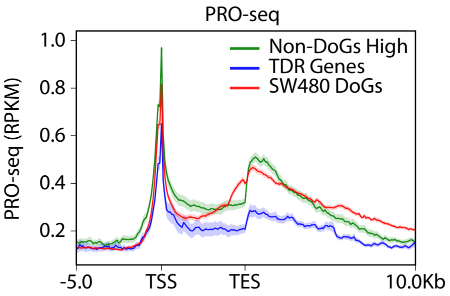

Fig. S5. Nascent Transcription Levels at TDR, Non-DoG, and SW480 DoG genes.

Metaplot of PRO-seq signal at highly expressed non-DoG genes (green), TDRs genes (blue), and SW480 DoG-producing genes (red) in SW480 cells. PRO-seq distribution (RPKM) spanning 5 kb upstream of the TSS to 10 kb downstream of the TES of the genes is represented.

Supplementary Tables

Table S1. List of DoG RNAs, DoG extension strengths, and differentially expressed DoG RNAs in BRCA, COAD, and LIHC tumors from TCGA

Table S2. List of differentially expressed stage-specific DoG RNAs in BRCA, COAD, and LIHC tumors from TCGA

Table S3. List of identified DoG RNAs, DoG extension strengths, and differentially expressed DoG RNAs in COAD tumors versus paired NTs.

Table S4. List of differentially expressed DoG RNAs and DoG producing host genes in paired COAD tumors versus NTs.

Table S5. List of identified DoG RNAs, DoG extension strengths, and differentially expressed DoG RNAs in normal and colon cancer cell lines.

Table S6. List of differentially expressed DoG RNAs and DoG producing host genes in normal and colon cancer cell lines.

Table S7. List of DoG RNAs identified in shCtrl, shTOP1, DMSO, and CPT treatment in SW480 cells

Table S8. The sequences of RT-qPCR primers, siRNAs, and shRNAs
